# Supplementary material for: Exosite inhibition of ADAMTS-5 by a glycoconjugated arylsulfonamide
Source: Sci Rep. 2021 Jan 13;11:949. doi: 10.1038/s41598-020-80294-1 (PMC7806935; doi:10.1038/s41598-020-80294-1)
Supplement: Supplementary file 1 — Supplementary Information 1. [file 41598_2020_80294_MOESM1_ESM.pdf]

SUPPLEMENTARY MATERIAL

Exosite inhibition of ADAMTS-5 by a glycoconjugated arylsulfonamide

Santamaria Salvatore[1]*, Doretta Cuffaro[2], Elisa Nuti[2]*, Lidia Ciccone,[2] Tiziano Tuccinardi,[2] Francesca Liva[2], Felicia D'Andrea,[2] Rens de Groot,[1,3] Armando Rossello[2] and Josefin Ahnström[1]

[1] Department of Immunology and Inflammation, Imperial College London, Du Cane Road, W12 0NN, London, United Kingdom
[2] Department of Pharmacy, University of Pisa, via Bonanno 6, 56126 Pisa, Italy.
[3] Institute of Cardiovascular Science, University College London, 51 Chenies Mews, WC1E 6HX, London, United Kingdom
Correspondence: Salvatore Santamaria s.santamaria@imperial.ac.uk; Elisa Nuti elisa.nuti@unipi.it
* Joint corresponding authors

Contents

Chemistry

Supplementary References

Supplementary Figures S1-S33

Supplementary Tables S1-S2

Chemistry

Instrumentation

Melting points were determined with a Kofler hot-stage apparatus and are uncorrected. ^{1}H NMR spectra were recorded in appropriate solvents with a Bruker Avance III HD 400 spectrometer operating at 400 MHz. ^{13}C NMR spectra were recorded with the above spectrometer operating at 100.57 MHz. The assignments were made, when possible, with the aid of DEPT, COSY, HSQC experiments. The first order proton chemical shifts (δ) are referenced to residual solvents and J-values are given in Hz. All reactions were followed by TLC on Kieselgel 60 F254 with detection by UV light and/or with ethanolic 10% phosphomolybdic or sulfuric acid, and heating. Kieselgel 60 (Merck, 230-400 mesh) was used for flash chromatography. Some chromatographic separations were conducted by using the automated system Isolera® Prime (Biotage), equipped with UV detector with variable wavelength (200-400 nm) or using prepacked ISOLUTE Flash Si II cartridges (Biotage). Microwave-assisted reactions were run in an Initiator+ (Biotage) microwave synthesizer. All reactions involving air- or moisture-sensitive reagents were performed under an argon atmosphere using anhydrous solvents. Anhydrous dimethylformamide (DMF), dichloromethane (CH_2Cl_2), 1,2-dichloethane (DCE) and THF were purchased from Sigma-Aldrich. $MgSO_4$ or Na_2SO_4 were used as the drying agents for solutions. Elemental analysis has been used to determine the purity of target compounds. Analytical results are within ± 0.40% of the theoretical values.

General strategy

The initially investigated compounds **1** and **2** (**Table 1**) were prepared as previously reported [1]. The new compounds **3a**, **b**, **4a-d**, **5b** and **6** tested in this study were synthesized as described in **Schemes 1-2**. For this, the alkynyl precursors **10a-d** were prepared as shown in **Scheme 1**. Sulfonyl chlorides **6b-d** [1, 2] were respectively converted into sulfonamides **7b-d**, by reaction with (+/-)-*sec*-butylamine in a mixture H_2O-dioxane (1:1 *v/v*) and triethylamine (TEA). The known carboxylic acid **8** [1] was protected by treatment with benzyl bromide and caesium carbonate to give benzyl ester **9.** Sulfonamides **7b-d** and **9** were *N*-alkylated by S_N2 reaction with propargyl bromide in DMF using potassium carbonate as base to afford alkynes **10a-d** in 57-100 % yields.

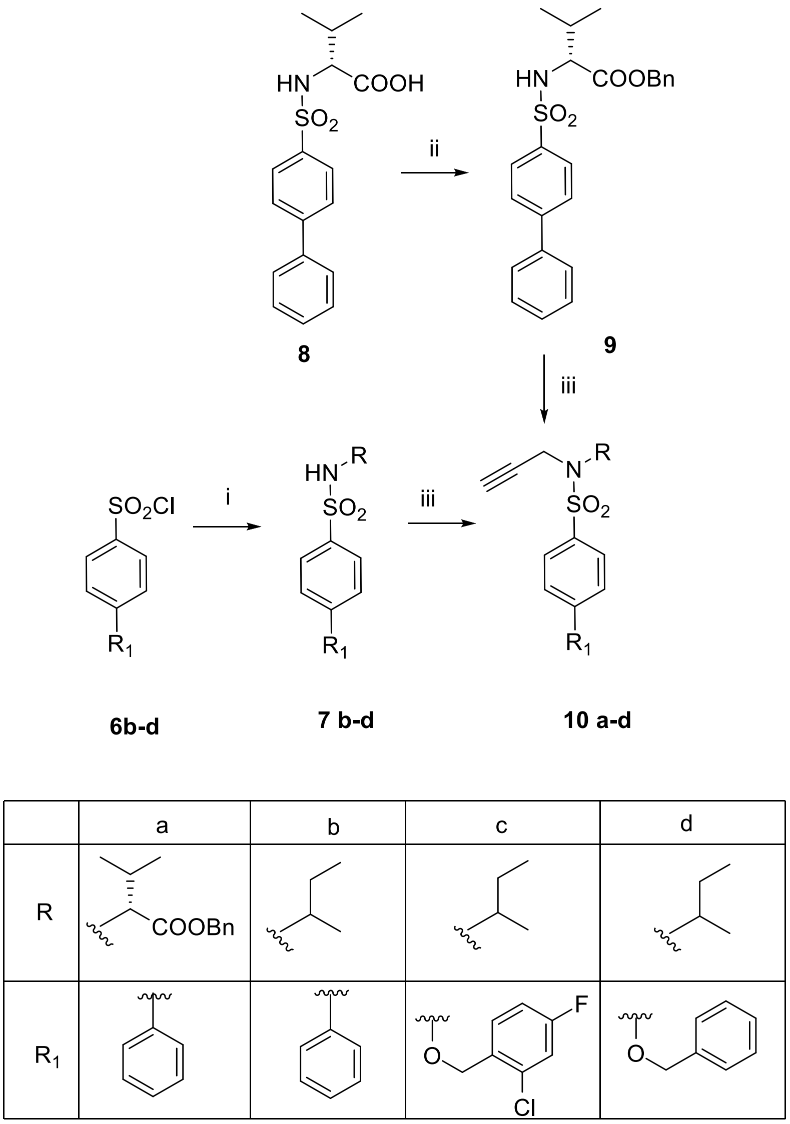

Scheme 1. Synthesis of alkynyl precursors **10a-d**. Reagents and conditions: i) (+/-)-*sec*-butylamine, Et_3N, 1:1 H_2O-Dioxane, 18 h (**7b**: quantitative yield; **7c**: quantitative yield; **7d**: 30%); ii) BnBr, Cs_2CO_3, DMF, 18 h (63%); iii) propargyl bromide, K_2CO_3, DMF, 48 h (**10a**: 94%; **10b**: quantitative yield; **10c**: 87%; **10d**: 57%).

The known 3-azidopropyl benzoate **18** [3] was prepared starting from 3-hydroxypropyl *p*-toluenesulfonate [4] (**Scheme 2**). The benzoylation of the hydroxyl portion was achieved by treatment with benzoyl chloride in DCM using DMAP and triethylamine as bases, to give tosylate **17**. 3-Azidopropyl benzoate **18** was obtained by conversion of **17** into the corresponding azide through an S_N2 reaction (NaN_3, DMF) in a nearly quantitative yield (98 %).

The β-glycosyl azides **11**, **12** and **13**, and the 3-azidopropyl benzoate **18** were conjugated to the proper alkynes **10a-d** (**Scheme 2**) by CuAAC click chemistry according to reported conditions [1]. The reactions were performed in a mixture DMF-H_2O (4:1 *v/v*) with copper(II) sulfate, sodium ascorbate catalytic system and heated under microwave irradiation at 80 °C for 30-45 min. Compound **6** was isolated by flash chromatography in good yield (79%). The desired 1,2,3-triazole derivatives **14a**, **14b**, **15a-d**, and **16b**, were used directly without further purification in the following reaction step**.**

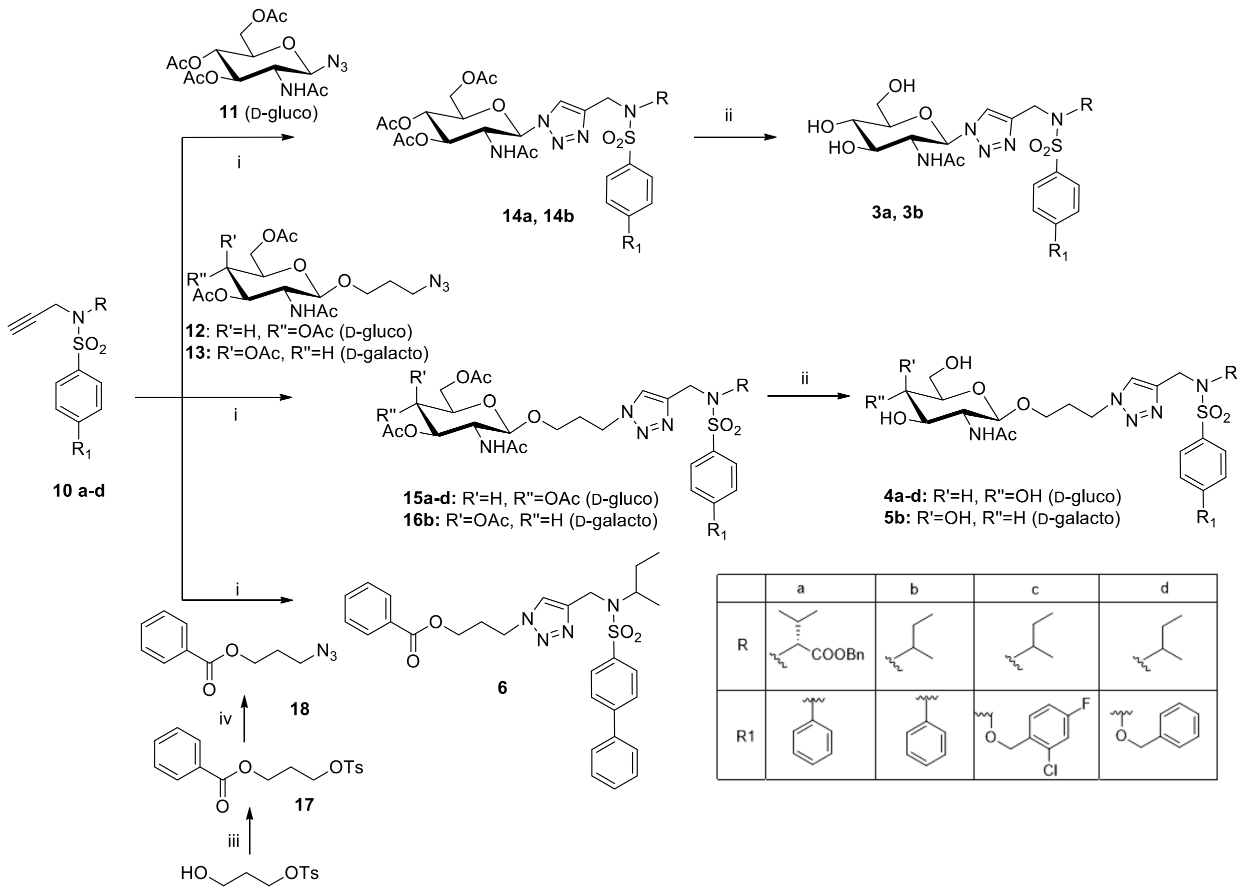

Scheme 2. Synthesis of 1,2,3-triazole glycoconjugates **3a**, **3b**, **4a-d**, **5b** and benzoyl derivative **6**. Reagents and conditions for **3a**, **3b**, **4a-d**, **and 5b**: i) $CuSO_4 \cdot 5H_2O$, sodium ascorbate, 4:1 DMF-H_2O, microwave, 80 °C, 30-45 min; ii) NH_3-MeOH 3.5N, 20-24 h. Yield over two steps: 29% for **3a**; 55% for **3b**; 50% for **4a**; 40% for **4b**; 14% for **4c**, 91% for **4d**, and 65% for **5b.** Reagents and conditions for **6**: i) $CuSO_4 \cdot 5H_2O$, sodium ascorbate, 4:1 DMF-H_2O, microwave, 80 °C, 45 min (79%); iii) BzCl, Et_3N, DMAP, DCM room temperature (RT) overnight (o/n) (56%); iv) NaN_3, DMF, 80°C, 3h (98%).

Finally, the *O*-deacetylation of **14a, 14b**, **15a-d and 16b** by treatment with NH_3-MeOH 3.5N afforded the deprotected derivatives **3a**, **3b** and **4a-d** in good yields (14-91 %). All compound structures were confirmed by mono- and two-dimensional NMR analyses (^{1}H, ^{13}C, COSY, HSQC) (**Supplementary Figures S5-S26**).

Synthesis of compounds 11- 13.

Compounds **11** [1], **12** [1], and **13** [5] were prepared as previously reported.

General Procedure for the Synthesis of Sulfonamides 7 b-d

To a solution of (+/-)-*sec*-butylamine (1 equiv.) in H_2O (0.8 mL/mmol) and dioxane (0.8 mL/mmol) containing TEA (2 equiv.), the proper sulfonyl chloride (1.2 equiv.) was added. The mixture was stirred at room temperature overnight. Dioxane was evaporated at diminished pressure, and the residue was taken up in H_2O and extracted with EtOAc (3×10 mL). Organic layers were collected,

dried over Na_2SO_4, filtered and concentrated in *vacuo*, affording the desired sulfonamides as yellowish solids.

N-[(+/-)-sec-butyl]-[1,1'-biphenyl]-4-sulfonamide (**7b**). The title compound was prepared from commercial biphenyl-4-sulfonyl chloride **6b** following the general procedure. Yellow solid (quantitative yield). ^{1}H NMR (400 MHz, $CDCl_3$) δ: 7.94 (m, 2H, Ar-*H*), 7.72 (m, 2H, Ar-*H*), 7.69 (m, 2H, Ar-*H*), 7.50-7.41 (m, 3H, Ar-*H*), 4.49 (bs, 1H, NH), 3.31 (m, 1H, C*H*CH_3), 1.43 (m, 2H, $CH_2$$CH_3$), 1.06 (d, 3H, J_{vic}=6.8 Hz, CHCH_3), 0.82 (t, 3H, J_{vic}=7.2 Hz, CH_2CH_3).

N-[(+/-)-sec-butyl]-4-[(2-chloro-4-fluorobenzyl)-oxy]-benzenesulfonamide (**7c**). The title compound was prepared from the known sulfonyl chloride **6c** [2] following the general procedure. Yellow solid (quantitative yield). ^{1}H NMR (400 MHz, $CDCl_3$) δ: 7.83 (m, 2H, Ar-*H*), 7.50 (dd, 1H, *J*=6.0 Hz, *J*=8.6 Hz, Ar-*H*), 7.19 (dd, 1H, *J*=8.4 Hz, *J*=2.6 Hz, Ar-*H*), 7.07-6.96 (m, 3H, Ar-*H*), 5.17 (s, 2H, CH_2O), 4.19 (d, 1H, *J*=8.0 Hz, NH), 3.28-3.23 (m, 1H, C*H*CH_3), 1.44-1.36 (m, 2H, C$H_2$$CH_3$), 1.04 (d, 3H, J_{vic}=6.4 Hz, CHCH_3), 0.80 (t, 3H, J_{vic}=7.2 Hz, CH_2CH_3).

4-(benzyloxy)-N-[(+/-)-sec-butyl]benzenesulfonamide (**7d**). The title compound was prepared from the known sulfonyl chloride **6d** [2] following the general procedure. The crude was purified by flash chromatography (5:1 *n*-hexane-EtOAc) using an Isolute Flash Si II cartridge to afford **7d** as a yellow solid (30% yield). ^{1}H NMR (400 MHz, $CDCl_3$) δ: 7.80 (m, 2H, Ar-*H*), 7.43-7.40 (m, 5H, Ar-*H*), 7.04 (m, 2H, Ar-*H*), 5.13 (s, 2H, CH_2O), 4.13 (d, 1H, *J*=8.4 Hz, NH), 3.25-3.22 (m, 1H, C*H*CH_3), 1.42-1.38 (m, 2H, C$H_2$$CH_3$), 1.03 (d, 3H, J_{vic}=6.4 Hz, CHCH_3), 0.80 (t, 3H, J_{vic}=7.2 Hz, CH_2CH_3).

(R)-benzyl 2-([1,1'-biphenyl]-4-ylsulfonamido)-3-methylbutanoate (**9**). To a solution of the known carboxylic acid **8** [1] (965 mg, 2.895 mmol) in dry DMF (3.8 mL), Cs_2CO_3 (707 mg, 2.171 mmol) was added at 0 °C under inert atmosphere (Ar). The mixture was stirred for 1 h at 0 °C and then benzyl bromide (0.26 mL, 2.171 mmol) was added. After stirring overnight at room temperature, the reaction mixture was taken up with water and extracted with EtOAc (3×125 mL). The collected organic layers were washed with brine, dried over Na_2SO_4, and concentrated at diminished pressure. The crude was purified by flash chromatography (8:1 *n*-hexane-EtOAc) affording the benzyl derivative **9** as a white solid (577 mg, 63% yield). ^{1}H NMR (400 MHz, $CDCl_3$) δ: 7.87 (m, 2H, Ar-*H*); 7.64 (m, 2H, Ar-*H*); 7.59-7.57 (m, 2H, Ar-*H*); 7.51-7.41 (m, 3H, Ar-*H*), 7.27-7.24 (m, 3H, Ar-*H*); 7.15- 7.13 (m, 2H, Ar-*H*), 5.16 (d, 1H, *J*=10.1 Hz, N*H*), 4.88, 4.83 (AB system, 2H, $J_{A,B}$=10.8 Hz, CH_2Ph), 3.84 (dd, 1H, *J*=4.9 Hz, C*H*N), 2.11-2.06 (m, 1H, CHCH_3), 0.98 (d, 3H, J_{vic}=6.8 Hz, CH_3), 0.86 (d, 3H, J_{vic}=6.8 Hz, CH_3).

General procedure for the synthesis of propargyl derivatives 10a-d

To a solution of the proper sulfonamide (1 equiv.) in dry DMF (1.6 mL/mmol) propargyl bromide (80% in toluene, 1.2 equiv.) and potassium carbonate (10 equiv.) were added. The resulting suspension was stirred at room temperature for 2 days under argon atmosphere. The mixture was diluted with water and extracted with EtOAc (3×30 mL). The combined organic phases were washed with brine, dried over Na_2SO_4 and the solvent was removed at diminished pressure. The crude product was purified by column chromatography on silica gel or by trituration to give the desired compounds **10a-d**.

(R)-Benzyl 3-methyl-2-(N-(prop-2-yn-1-yl)-[1,1'-biphenyl]-4-ylsulfonamido)butanoate (**10a**)
The title compound was prepared from sulfonamide **9** following the general procedure. The crude product was purified by trituration with *n*-hexane to afford **10a** as a yellow solid (94% yield). ^{1}H NMR (400 MHz, $CDCl_3$) δ: 7.94-7.88 (m, 2H, Ar-*H*), 7.60-7.55 (m, 4H, Ar-*H*), 7.50-7.40 (m, 3H, Ar-*H*), 7.29-7.26 (m, 3H, Ar-*H*), 7.22-7.19 (m, 2H, Ar-*H*), 5.00, 4.84 (AB system, 2H $J_{A,B}$=12.4 Hz, CH_2Ph), 4.40 (dd, 1H, J_{gem}=21.2 Hz, *J*=2.4 Hz, CH_2C≡), 4.18 (dd, 1H, CH_2C≡), 4.21-4.05 (m, 1H, C*H*N), 2.26-2.22 (m, 1H, C*H*Me_2), 2.14 (t, 1H, *J*=2.4 Hz, C≡C*H*), 1.07 (d, 3H, J_{vic}=6.8 Hz, CH_3), 0.95 (d, 3H, J_{vic}=6.8 Hz, CH_3).

N-[(+/-)-sec-butyl]-N-(prop-2-yn-1-yl)-[1,1'-biphenyl]-4-sulfonamide (**10b**). The title compound was prepared from sulfonamide **7b** following the general procedure. The crude product was purified by trituration with *n*-hexane to afford **10b** as a yellow solid (quantitative yield). ^{1}H NMR (400 MHz, $CDCl_3$) δ: 7.96 (m, 2H, Ar-*H*), 7.70 (m, 2H, Ar-*H*), 7.62 (m, 2H, Ar-*H*), 7.48 (m, 2H, Ar-*H*), 7.45-7.40 (m, 1H, Ar-*H*), 4.10 (dd, 1H, J_{gem}=18.4 Hz, *J*=2.4 Hz, CH_2C≡), 4.00 (dd, 1H, CH_2C≡), 3.92-3.87 (m, 1H, C*H*CH_3), 2.15 (t, 1H, *J*=2.4 Hz, C≡C*H*), 1.61-1.47 (m, 2H, C$H_2$$CH_3$), 1.11 (d, 3H, J_{vic}=6.8 Hz, CHCH_3), 0.85 (t, 3H, J_{vic}=7.2 Hz, CH_2CH_3).

N-[(+/-)-sec-butyl]-4-[(2-chloro-4-fluorobenzyl)oxy]-N-(prop-2-yn-1-yl)benzenesulfonamide (**10c**). The title compound was prepared from sulfonamide **7c** following the general procedure. The crude product was purified by trituration with *n*-hexane to afford **10c** as a yellow solid (87% yield). ^{1}H NMR (400 MHz, $CDCl_3$) δ: 7.86 (m, 2H, Ar-*H*), 7.50 (dd, 1H, *J*=5.9 Hz, *J*=8.4 Hz, Ar-*H*), 7.18 (dd, 1H, *J*=8.4 Hz, *J*=2.5 Hz, Ar-*H*), 7.06-7.00 (m, 3H, Ar-*H*), 5.17 (s, 2H, CH_2O), 4.10 (dd, 1H, J_{gem}=18.5 Hz, *J*= 2.4 Hz, CH_2C≡), 4.05 (dd, 1H, CH_2C≡), 3.86-3.80 (m, 1H, C*H*CH_3), 2.15 (t, 1H, *J*=2.4 Hz, C≡C*H*), 1.61-1.45 (m, 2H, C$H_2$$CH_3$), 1.09 (d, 3H, J_{vic}=6.4 Hz, CHCH_3), 0.83 (t, 3H, J_{vic}=7.2 Hz, CH_2CH_3).

4-(Benzyloxy)- N-[(+/-)-sec-butyl]-N-(prop-2-yn-1-yl)benzenesulfonamide (**10d**). The title compound was prepared from sulfonamide **7d** following the general procedure. The crude product was purified by flash chromatography (10:1 *n*-hexane-EtOAc) using an Isolute Flash Si II cartridge to afford **10c** as a yellow solid (57% yield). ^{1}H NMR (400 MHz, $CDCl_3$) δ: 7.83 (m, 2H, Ar-*H*), 7.43-7.34 (m, 5H, Ar-*H*), 7.02 (m, 2H, Ar-*H*), 5.12 (s, 2H, CH_2O), 4.09 (dd, 1H, J_{gem}=18.4 Hz, *J*= 2.4 Hz, CH_2C≡), 4.04 (dd, 1H, CH_2C≡), 3.85-3.80 (m, 1H, C*H*CH_3), 2.11 (t, 1H, *J*=2.4 Hz, C≡C*H*), 1.57-1.45 (m, 2H, C$H_2$$CH_3$), 1.09 (d, 3H, J_{vic}=6.8 Hz, CHCH_3), 0.83 (t, 3H, J_{vic}=7.2 Hz, CH_2CH_3).

General procedure for the synthesis of glycoconjugates 3a, 3b, 4a-d, 5b and benzoyl derivative 6. The appropriate alkyne (1 equiv.) and the opportune azide (1.1 equiv.) were dissolved in a mixture of 4:1 DMF-H_2O (24 mL/ mmol) in the presence of $CuSO_4 \cdot 5H_2O$ (1.5 equiv.) and sodium ascorbate (3 equiv.). The solution was heated under microwave irradiation to 80 °C for 30-45 min, then diluted with Et_2O (12 mL) and washed with saturated aq NH_4Cl (25 mL). The organic phase was separated and the aq layer extracted with Et_2O (2×25 mL). The collected organic extracts were dried (Na_2SO_4), filtered and concentrated under diminished pressure. Flash chromatographic purification over silica gel of the crude product gave triazole-linked derivative **6** pure as a white solid. The acetylated glycoconjugates **14a**, **14b, 15a-d** and **16b** were used in the following step without any

purification. A solution of the appropriate acetylated glycoconjugate (**14a**, **14b**, **15a-d**, or **16b**, 0.1 mmol) in MeOH (1.0 mL) was treated with NH_3-MeOH 7N (1.0 mL) and the solution was stirred at room temperature until the starting compound was completely reacted (TLC, 8:2 $CHCl_3$-MeOH, 20-24 h). The solution was co-evaporated with toluene (4×10 mL) under diminished pressure. Flash chromatographic purification over silica gel of the crude product gave pure triazole-linked derivatives (**3a**, **3b**, **4a-d**, and **5b**) as white solids.

*Glycoconjugate **3a***. The title compound was obtained from alkyne **10a** and azide **11** (D-gluco) following the general procedure. After treatment, the glycoconjugate **14a** was isolated and used directly in the basic hydrolysis with NH_3-MeOH 3.5N. The crude was purified by flash chromatography (20:1 $CHCl_3$-MeOH) using an Isolute Flash Si II cartridge to afford **3a** as a white solid (29% yield from **10a**); mp 114-116 °C; ^{1}H NMR (400 MHz, CD_3OD) δ: 8.18 (s, 1H, Ar-*H* triazole), 7.92-7.80 (m, 2H, Ar-*H*), 7.70-7.63 (m, 4H, Ar-*H*), 7.51-7.37 (m, 3H, Ar-*H*), 7.25-7.18 (m, 5H, Ar-*H*), 5.79 (d, 1H, $J_{1,2}$=9.8 Hz, H-1), 4.89, 4.72 (AB system, 2H, $J_{A,B}$=12.2 Hz, $PhCH_2O$), 4.84, 4.66 (AB system, 2H, $J_{A,B}$=16.6 Hz, CH_2NSO_2), 4.23 (bt, 1H, $J_{1,2}$=$J_{2,3}$=9.8 Hz, H-2), 4.09 (d, 1H, J_{vic}=10.6 Hz, C*H*N), 3.91 (bd, 1H, $J_{6a,6b}$=12.1 Hz, H-6b), 3.78-3.67 (m, 2H, H-3, H-5), 3.63-3.51 (m, 2H, H-4, H-6a), 2.29 (m, 1H, C*H*Me_2), 1.79 (s, 3H, CH_3CON), 0.85 (d, 3H, J_{vic}=6.5 Hz, CH_3), 0.71 (d, 3H, J_{vic}=6.5 Hz, CH_3); ^{13}C NMR (100 MHz, CD_3OD) δ: 173.4, 171.7 (2×C=O), 147.0 (Ar-*C*-SO_2, *C*-triazole), 140.3, 139.2, 136.5 (3×Ar-*C*), 1130.2-125.3 (Ar-*C*H), 125.3 (CH-triazole), 88.2 (C-1), 81.4 (C-5), 75.8 (C-3), 71.4 (C-4), 67.7 (OCH_2Ph), 67.5 (CHN), 62.4 (C-6), 56.9 (C-2), 40.9 (CH_2NSO_2), 30.2 (*C*HMe_2), 22.6 (MeCON), 20.3, 19.5 (*Me*$_2$CH). Elemental analysis calcd (%) for $C_{35}H_{41}N_5O_9S$: C 59.39, H 5.84, N 9.89; found: C 59.42, H 5.90, N 9.93.

*Glycoconjugate **3b***. The title compound was obtained from alkyne **10b** and azide **11** (D-gluco) following the general procedure. After treatment, the glycoconjugate **14b** was isolated and used directly in the basic hydrolysis with NH_3-MeOH 3.5N. The crude was purified by flash chromatography (20:1 $CHCl_3$-MeOH) using an Isolute Flash Si II cartridge to afford **3b** as a white solid (55% yield from **10b**); mp 211-213 °C. The NMR analysis of **3b** (CD_3OD) showed a mixture of the two diastereoisomeric forms in the ratio of 1:1, measured on the relative intensities of the H-1 signals at δ 5.79 and 5.78 respectively. ^{1}H NMR (400 MHz, CD_3OD) *one diastereoisomeric form* δ: 8.08 (s, 1H, Ar-*H* triazole), 5.79 (d, 1H, $J_{1,2}$=9.8 Hz, H-1), 4.52, 4.40 (AB system, 2H, $J_{A,B}$=16.6 Hz, CH_2NSO_2), 1.78 (s, 3H, CH_3CONH), 1.57-1.42 (m, 2H, CH_3CH_2), 0.99 (d, 3H, J_{vic}=6.7 Hz, CH_3CH), 0.72 (t, 3H, J_{vic}=7.4 Hz, CH_3CH_2); *other diastereoisomeric form* δ: 8.07 (s, 1H, Ar-*H* triazole), 5.78 (d, 1H, $J_{1,2}$=9.8 Hz, H-1), 4.48, 4.43 (AB system, 2H, $J_{A,B}$=17.1 Hz, CH_2NSO_2), 1.77 (s, 3H, CH_3CONH), 1.41-1.31 (m, 2H, CH_3CH_2), 0.98 (d, 3H, J_{vic}=6.7 Hz, CH_3CH), 0.70 (t, 3H, J_{vic}=7.4 Hz, CH_3CH_2); *cluster of signals for both diastereoisomeric forms* δ: 7.93-7.90 (m, 2H, Ar-*H*), 7.84-7.82 (m, 2H, Ar-*H*), 7.71-7.69 (m, 2H, Ar-*H*), 7.51-7.47 (m, 2H, Ar-*H*), 7.43-7.39 (m, 1H, Ar-*H*), 4.25-4.19 (m, 1H, C*H*N), 3.92-3.84 (m, 2H, H-2, H-6b), 3.76-3.71 (m, 1H, H-6a), 3.68 (bt,1H, $J_{2,3}$= $J_{3,4}$=9.3 Hz, H-3); 3.60-3.51 (m, 2H, H-4, H-5). ^{13}C NMR (100 MHz, CD_3OD) *one diastereoisomeric form* δ: 124.8 (*C*H-triazole), 88.4 (C-1), 76.2 (C-3), 57.8 (*C*HN), 57.0 (C-2), 38.9 (*C*H_2NSO_2), 29.4 (CH_3CH_2), 22.9 (*C*H_3CON), 19.1 (*C*H_3CH), 11.9 (*C*H_3CH_2); *other diastereoisomeric form* δ: 124.6 (*C*H-triazole), 88.3 (C-1), 76.1 (C-3), 57.7 (*C*HN), 56.9 (C-2), 38.8 (*C*H_2NSO_2), 29.3 (CH_3CH_2), 22.8 (*C*H_3CON), 18.9 (*C*H_3CH), 11.8 (*C*H_3CH_2); *cluster of signals for both diastereoisomeric forms* δ: 173.5 (C=O), 147.3-147.0 (Ar-*C*-SO_2, *C*-triazole), 141.1-140.8

(2×Ar-*C*), 130.4-128.5 (Ar-*C*H), 81.6 (C-5), 71.6 (C-4), 62.6 (C-6). Elemental analysis calcd (%) for $C_{27}H_{35}N_5O_7S$: C 56.53, H 6.15, N 12.21; found: C 56.55, H 6.17, N 12.23.

*Glycoconjugate **4a**.* The title compound was obtained from alkyne **10a** and azide **12** (D-gluco) following the general procedure. After treatment, the glycoconjugate **15a** was isolated and used directly in the basic hydrolysis with NH_3-MeOH 3.5N. The crude was purified by flash chromatography (20:1 $CHCl_3$-MeOH) using an Isolute Flash Si II cartridge to afford **4a** as a white solid (50% yield from **10a**); mp 88-90 °C; ^{1}H NMR (400 MHz, CD_3OD) δ: 7.89 (s, 1H, Ar-*H* triazole), 7.84-7.81 (m, 2H, Ar-*H*), 7.67-7.63 (m, 4H, Ar-*H*), 7.52-7.43 (m, 3H, Ar-*H*), 7.27-7.00 (m, 5H, Ar-*H*), 4.94, 4.81(AB system, 2H, $J_{A,B}$= 12.2 Hz, CH_2Ph), 4.82, 4.66 (AB system, 2H, $J_{A,B}$=16.6 Hz, CH_2NSO$_2$), 4.50-4.40 (m, 2H, CH_2N), 4.36 (d, 1H, $J_{1,2}$=8.4 Hz, H-1), 4.16 (d, 1H, J_{vic}=10.6 Hz, C*H*N), 3.92-3.82 (m, 2H, H-6b, ½CH_2O), 3.73-3.66 (m, 2H, H-2, H-6a), 3.48-3.33 (m, 3H, H-3, H-4, ½CH_2O), 3.26 (ddd, 1H, $J_{5,6b}$=2.6 Hz, $J_{5,6a}$=6.0 Hz, $J_{4,5}$=10.0 Hz, H-5), 2.08-2.02 (m, 2H, CH_2), 2.27-2.25 (m, 1H, C*H*CH_3), 2.01 (s, 3H, CH_3CONH), 0.87 (d, 3H, *J*=6.5 Hz, CH_3), 0.77 (d, 3H, *J*=6.5 Hz, CH_3); ^{13}C NMR (100 MHz, CD_3OD) δ:173.9, 171.9 (2×C=O), 147.1 (Ar-*C*-SO$_2$, *C*-triazole), 140.4, 139.7, 136.8 (3×Ar-*C*), 130.4-128.5 (Ar-*C*H), 126.5 (*C*H-triazole), 103.1 (C-1), 78.2 (C-5), 76.2 (C-3), 72.3 (C-4), 68.0 (PhCH_2O), 67.5 (CHN), 66.8 (CH_2O), 63.0 (C-6), 57.6 (C-2), 48.4 (CH_2N), 41.3 (CH_2NSO$_2$), 31.8 (CH_2), 30.2 (Me$_2$*C*H), 23.4 (*C*H_3CON), 20.4, 19.8 (*Me*$_2$CH). Elemental analysis calcd (%) for $C_{38}H_{47}N_5O_{10}S$: C 59.59, H 6.19, N 9.14; found: C 59.61, H 6.17, N 9.20.

*Glycoconjugate **4b**.* The title compound was obtained from alkyne **10b** and azide **12** (D-gluco) following the general procedure. After treatment, the glycoconjugate **15b** was isolated and used directly in the basic hydrolysis with NH_3-MeOH 3.5N. The crude was purified by flash chromatography (20:1 $CHCl_3$-MeOH) using an Isolute Flash Si II cartridge to afford **4b** as a white solid (40% yield from **10b**); mp 73-75 °C. The NMR analysis of **4b** (CD_3OD) showed a mixture of the two diastereoisomeric forms in the ratio of 1:1, measured on the relative intensities of the CH_3CH signals at δ 1.01 and 1.00 respectively. ^{1}H NMR (400 MHz, CD_3OD) *one diastereoisomeric form* δ: 7.98 (s, 1H, Ar-*H* triazole), 2.03 (s, 3H, CH_3CONH), 1.55-1.45 (m, 2H, CH_3CH_2), 1.01 (d, 3H, J_{vic}=6.7 Hz, CH_3CH), 0.71 (t, 3H, J_{vic}=7.3 Hz, CH_3CH$_2$); *other diastereoisomeric form* δ: 7.97 (s, 1H, Ar-*H* triazole), 2.02 (s, 3H, CH_3CONH), 1.44-1.35 (m, 2H, CH_3CH_2), 1.00 (d, 3H, J_{vic}=6.7 Hz, CH_3CH), 0.70 (t, 3H, J_{vic}=7.3 Hz, CH_3CH$_2$); *cluster of signals for both diastereoisomeric forms* δ: 7.93-7.90 (m, 2H, Ar-*H*), 7.84-7.82 (m, 2H, Ar-*H*), 7.71-7.69 (m, 2H, Ar-*H*), 7.51-7.48 (m, 2H, Ar-*H*), 7.44-7.40 (m, 1H, Ar-*H*), 4.56-4.38 (m, 4H, CH_2N, CH_2NSO$_2$), 4.36 (d, 1H, $J_{1,2}$=8.4 Hz, H-1), 3.90-3.80 (m, 3H, H-6b, C*H*N, ½CH_2O), 3.73-3.66 (m, 2H, H-2, H-6a), 3.45 (bt, 1H, $J_{2,3}$=$J_{3,4}$=9.5 Hz, H-3); 3.39-3.27 (m, 2H, H-4, ½CH_2O), 3.26 (ddd, 1H, $J_{5,6b}$=1.7 Hz, $J_{5,6a}$= 5.2 Hz, $J_{4,5}$=10.2 Hz, H-5), 2.17-2.08 (m, 2H, CH_2). ^{13}C NMR (100 MHz, CD_3OD) *one diastereoisomeric form* δ: 173.9 (C=O), 126.2 (*C*H-triazole), 66.5 (*C*H$_2$O), 57.6 (C-2), 31.6 (*C*H$_2$), 29.2 (CH_3*C*H$_2$), 18.9 (*C*H$_3$CH), 11.7 (*C*H$_3$CH$_2$); *other diastereoisomeric form* δ: 173.8 (C=O), 126.1 (*C*H-triazole), 66.4 (*C*H$_2$O), 57.5 (C-2), 31.5 (*C*H$_2$), 29.1 (CH_3*C*H$_2$), 18.7 (*C*H$_3$CH), 11.5 (*C*H$_3$CH$_2$); *cluster of signals for both diastereoisomeric forms* δ: 147.0-146.8 (Ar-*C*-SO$_2$, *C*-triazole), 140.9-140.5 (2×Ar-*C*), 130.2-128.3 (Ar-*C*H), 102.8 (C-1), 78.0 (C-5), 76.0 (C-3), 72.1 (C-4), 62.8 (C-6), 57.4 (*C*HN), 48.1 (*C*H$_2$N), 38.7 (*C*H$_2$NSO$_2$), 23.1 (*C*H$_3$CON). Elemental analysis calcd (%) for $C_{30}H_{41}N_5O_8S$: C 57.04, H 6.54, N 11.09; found: C 57.07, H 6.57, N 11.12.

Glycoconjugate ***4c***. The title compound was obtained from alkyne **10c** and azide **12** (D-gluco) following the general procedure. After treatment, the glycoconjugate **15c** was isolated and used directly in the basic hydrolysis with NH_3-MeOH 3.5N. The crude was purified by flash chromatography ($CHCl_3$-MeOH 95:1) using Biotage Isolera (5 g Zip Sphere Column) to afford **4c** as a white solid (14% yield from **10c**); mp 100-102 °C. The NMR analysis of **4c** (CD_3OD) showed a mixture of the two diastereoisomeric forms in the ratio of 1:1, measured on the relative intensities of the CH_3CH signals at δ 0.97 and 0.96 respectively. ^1H NMR (400 MHz, CD_3OD) *one diastereoisomeric form* δ: 7.95 (s, 1H, Ar-*H* triazole), 4.44, 4.38 (AB system, 2H, $J_{A,B}$=16.3 Hz, CH_2NSO_2), 2.03 (s, 3H, CH_3CONH), 0.97 (d, 3H, J_{vic}=6.7 Hz, CH_3CH), 0.67 (t, 3H, J_{vic}=7.3 Hz, CH_3CH_2); *other diastereoisomeric form* δ: 7.94 (s, 1H, Ar-*H* triazole), 4.42, 4.40 (AB system, 2H, $J_{A,B}$= 16.5 Hz, CH_2NSO_2), 2.02 (s, 3H, CH_3CONH), 0.96 (d, 3H, J_{vic}=6.7 Hz, CH_3CH), 0.66 (t, 3H, J_{vic}=7.3 Hz, CH_3CH_2); *cluster of signals for both diastereoisomeric forms* δ: 7.80 (m, 2H, Ar-*H*), 7.60 (dd, 1H, *J*=6.0 Hz, *J*=8.4 Hz, Ar-*H*), 7.30 (dd, 1H, *J*=2.6 Hz, *J*=8.4 Hz, Ar-*H*), 7.17 (m, 2H, Ar-*H*), 7.12 (dd, 1H, *J*=2.6 Hz, *J*=8.4 Hz, Ar-*H*), 5.23 (s, 2H, $PhCH_2O$), 4.48 (bt, 2H, J_{vic}=6.6 Hz, CH_2N), 4.38 (d, 1H, $J_{1,2}$=8.4 Hz, H-1), 3.93-3.85 (m, 2H, H-6b, ½CH_2O), 3.79 (m, 1H, C*H*N), 3.73-3.67 (m, 2H, H-2, H-6a), 3.46 (bt, 1H, $J_{2,3}$=$J_{3,4}$=9.3 Hz, H-3); 3.40-3.31 (m, 2H, H-4, ½CH_2O), 3.26 (ddd, 1H, $J_{5,6b}$=2.0 Hz, $J_{5,6a}$=5.6 Hz, $J_{4,5}$=9.6 Hz, H-5), 2.18-2.08 (m, 2H, CH_2), 1.50-1.32 (m, 2H, CH_3CH_2). ^{13}C NMR (100 MHz, CD_3OD) *one diastereoisomeric form* δ: 173.9 (C=O), 126.1 (*C*H-triazole), 66.4 (CH_2O), 57.4 (C-2), 31.5 (CH_2), 29.1 (CH_3CH_2), 18.8 (CH_3CH), 11.6 (CH_3CH_2); *other diastereoisomeric form* δ: 173.8 (C=O), 126.0 (*C*H-triazole), 66.3 (CH_2O), 57.3 (C-2), 31.4 (CH_2), 29.0 (CH_3CH_2), 18.6 (CH_3CH), 11.5 (CH_3CH_2); *cluster of signals for both diastereoisomeric forms* δ: 163.3 (Ar-*C*O), 147.4-147.3 (Ar-*C*-SO_2, *C*-triazole), 135.5, 134.5, 131.6 (Ar-*C*), 132.6-130.4 (Ar-*C*H), 118.0-115.2 (Ar-*C*H), 102.8 (C-1), 78.0 (C-5), 76.0 (C-3), 72.1 (C-4), 68.3 ($PhCH_2O$), 62.8 (C-6), 57.3 (*C*HN), 23.1 (CH_3CON), 48.1 (CH_2N), 38.6 (CH_2NSO_2), Elemental analysis calcd (%) for $C_{31}H_{41}ClFN_5O_9S$: C 52.13, H 5.79, N 9.81; found: C 52.16, H 5.77, N 9.83.

Glycoconjugate ***4d***. The title compound was obtained from alkyne **10d** and azide **12** (D-gluco) following the general procedure. After treatment, the glycoconjugate **15d** was isolated and used directly in the basic hydrolysis with NH_3-MeOH 3.5N. The crude was purified by flash chromatography (20:1 $CHCl_3$-MeOH) using an Isolute Flash Si II cartridge to afford **4d** as a white solid (91% yield from **10d**); mp 98-100 °C. The NMR analysis of **4d** (CD_3OD) showed a mixture of the two diastereoisomeric forms in the ratio of 1:1, measured on the relative intensities of the CH_3CH signals at δ 0.96 and 0.95 respectively. ^1H NMR (400 MHz, CD_3OD) *one diastereoisomeric form* δ: 7.94 (s, 1H, Ar-*H* triazole), 4.43, 4.37 (AB system, 2H, $J_{A,B}$=15.1 Hz, CH_2NSO_2), 2.03 (s, 3H, CH_3CONH), 0.96 (d, 3H, J_{vic}=6.7 Hz, CH_3CH), 0.67 (t, 3H, J_{vic}=7.3 Hz, CH_3CH_2); *other diastereoisomeric form* δ: 7.93 (s, 1H, Ar-*H* triazole), 4.41, 4.39 (AB system, 2H, $J_{A,B}$=15.0 Hz, CH_2NSO_2), 2.02 (s, 3H, CH_3CONH), 0.95 (d, 3H, J_{vic}=6.7 Hz, CH_3CH), 0.66 (t, 3H, J_{vic}=7.3 Hz, CH_3CH_2); *cluster of signals for both diastereoisomeric forms* δ: 7.79-7.70 (m, 2H, Ar-*H*), 7.45-7.33 (m, 5H, Ar-*H*), 7.19-7.10 (m, 2H, Ar-*H*), 5.18 (s, 2H, $PhCH_2O$), 4.48 (bt, 2H, J_{vic}=6.5 Hz, CH_2N), 4.45-4.35 (m, 1H, C*H*N), 4.38 (d, 1H, $J_{1,2}$=8.5 Hz, H-1), 3.94-3.86 (m, 2H, H-6b, ½CH_2O), 3.74-3.61 (m, 2H, H-2, H-6a), 3.46 (bt, 1H, $J_{2,3}$=$J_{3,4}$=9.5 Hz, H-3); 3.40-3.31 (m, 2H, H-4, ½CH_2O), 3.26 (ddd, 1H, $J_{5,6b}$=2.0 Hz, $J_{5,6a}$=5.7 Hz, $J_{4,5}$=9.6 Hz, H-5), 2.15-2.08 (m, 2H, CH_2), 1.48-1.33 (m, 2H, CH_3CH_2). ^{13}C NMR (100 MHz, CD_3OD) *one diastereoisomeric form* δ: 126.1

(*C*H-triazole), 66.4 (*C*H_2O), 62.8 (C-6), 57.4 (C-2), 31.5 (*C*H_2), 29.1 (CH_3*C*H_2), 18.8 (*C*H_3CH), 11.6 (*C*$H_3$$CH_2$); *other diastereoisomeric form* δ: 126.0 (*C*H-triazole), 66.3 (*C*H_2O), 62.7 (C-6), 57.3 (C-2), 31.4 (*C*H_2), 29.0 (CH_3*C*H_2), 18.6 (*C*H_3CH), 11.5 (*C*$H_3$$CH_2$); *cluster of signals for both diastereoisomeric forms* δ: 173.9 (C=O), 163.6 (Ar-*C*O), 137.8 (Ar-*C*-SO_2, *C*-triazole), 134.0 (Ar-*C*), 130.3-128.7 (Ar-*C*H), 116.3 (Ar-*C*H), 102.8 (C-1), 78.0 (C-5), 76.0 (C-3), 72.1 (C-4), 71.4 (Ph*C*H_2O), 23.1 (*C*H_3CON), 57.2 (*C*HN), 48.1 (*C*H_2N), 38.6 (*C*H_2NSO_2). Elemental analysis calcd (%) for $C_{31}H_{43}N_5O_9S$: C 56.26, H 6.55, N 10.58; found: C 56.29, H 6.57, N 10.60.

Glycoconjugate ***5b***. The title compound was obtained from alkyne **10d** and azide **13** (D-*galacto*) following the general procedure. After treatment, the glycoconjugate **16b** was isolated and used directly in the basic hydrolysis with NH_3-MeOH 3.5N. The crude was purified by flash chromatography (20:1 $CHCl_3$-MeOH) using an Isolute Flash Si II cartridge to afford **5b** as a white solid (91% yield from **10d**); mp 112-114 °C. The NMR analysis of **5b** (CD_3OD) showed a mixture of the two diastereoisomeric forms in the ratio of 1:1, measured on the relative intensities of the C*H*$_3$CH signals at δ 1.01 and 1.00 respectively. ^{1}H NMR (400 MHz, CD_3OD) *one diastereoisomeric form* δ: 7.97 (s, 1H, Ar-*H* triazole), 3.61 (dd, 1H, $J_{2,3}$=10.6 Hz, $J_{3,4}$=0.98 Hz, H-3), 2.04 (s, 3H, C*H*$_3$CONH), 1.53-1.48 (m, 2H, CH_3C*H*$_2$), 1.01 (d, 3H, J_{vic}=6.8 Hz, C*H*$_3$CH), 0.70 (t, 3H, J_{vic}=7.3 Hz, C*H*$_3$$CH_2$); *other diastereoisomeric form* δ: 7.96 (s, 1H, Ar-*H* triazole), 3.60 (dd, 1H, $J_{2,3}$=10.6 Hz, $J_{3,4}$=0.98 Hz, H-3), 2.03 (s, 3H, C*H*$_3$CONH), 1.47-1.37 (m, 2H, CH_3C*H*$_2$), 1.00 (d, 3H, J_{vic}=6.8 Hz, C*H*$_3$CH), 0.69 (t, 3H, J_{vic}=7.3 Hz, C*H*$_3$$CH_2$); *cluster of signals for both diastereoisomeric forms* δ: 7.93-7.89 (m, 2H, Ar-*H*), 7.83-7.80 (m, 2H, Ar-*H*), 7.70-7.67 (m, 2H, Ar-*H*), 7.51-7.47 (m, 2H, Ar-*H*), 7.44-7.39 (m, 1H, Ar-*H*), 4.55-4.40 (m, 4H, C*H*$_2$N, C*H*$_2$NSO_2), 4.34 (d, 1H, $J_{1,2}$=8.4 Hz, H-1), 4.01-3.96 (m, 1H, H-2), 3.90-3.87 (m, 3H, H-4, C*H*N, ½C*H*$_2$O), 3.85-3.73 (m, 2H, H-6a, H-6b), 3.51-3.48 (m, 1H, H-5), 3.41-3.35 (m, 1H, ½C*H*$_2$O), 2.15-2.08 (m, 2H, C*H*$_2$). ^{13}C NMR (100 MHz, CD_3OD) *one diastereoisomeric form* δ: 126.2 (*C*H-triazole), 66.4 (*C*H_2O), 29.2 (CH_3*C*H_2), 18.9 (*C*H_3CH); *other diastereoisomeric form* δ: 126.1 (*C*H-triazole), 66.3 (*C*H_2O), 29.1 (CH_3*C*H_2), 18.7 (*C*H_3CH); *cluster of signals for both diastereoisomeric forms* δ: 174.2 (C=O), 147.1-146.8 (Ar-*C*-SO_2, *C*-triazole), 140.9-140.4 (2×Ar-*C*), 130.2-128.3 (Ar-*C*H), 103.2 (C-1), 76.7 (C-5), 73.2 (C-3), 69.6 (C-4), 62.5 (C-6), 57.5 (*C*HN), 54.3 (C-2), 48.1 (*C*H_2N), 38.7 (*C*H_2NSO_2), 31.6 (*C*H_2), 23.2 (*C*H_3CON), 11.6 (*C*$H_3$$CH_2$). Elemental analysis calcd (%) for $C_{31}H_{43}N_5O_9S$: C 56.26, H 6.55, N 10.58; found: C 56.29, H 6.57, N 10.60.

Benzoyl derivative ***6***. The title compound was prepared from alkyne **10b** and azide **18** following the general procedure. After a Flash chromatography using Biotage Isolera (10 g Zip Sphere Column, 1:1 *n*-hexane-EtOAc) the benzoyl derivative **5** was isolated as a colourless oil (79% yield). ^{1}H NMR (400 MHz, $CDCl_3$) δ: 8.08-8.06 (m, 2H, Ar-*H*), 7.91-7.88 (m, 5H, Ar-*H*), 7.87 (s, 1H, Ar-*H* triazole), 7.75-7.73 (m, 2H, Ar-*H*), 7.64-7.60 (m, 5H, Ar-*H*), 7.52-7.43 (m, 5H, Ar-*H*), 4.56 (t, 2H, J_{vic}=7.1 Hz, C*H*$_2$N), 4.51-4.41(m, 2H, C*H*$_2$NSO_2), 4.37 (t, 2H, J_{vic}=6.0 Hz, C*H*$_2$O), 3.92-3.87 (m, 1H, C*H*N), 2.47-2.40 (m, 2H, C*H*$_2$), 1.55-1.46, 1.45-1.35 (2m, each 1H, CH_3C*H*$_2$), 1.02 (d, 3H, J_{vic}=6.8 Hz, C*H*$_3$CH), 0.71 (t, 3H, J_{vic}=7.3 Hz, C*H*$_3$$CH_2$); ^{13}C NMR (100 MHz, $CDCl_3$) δ: 166.3 (C=O), 146.6, 145.4 (Ar-*C*-SO_2, *C*-triazole), 139.5, 139.2, 133.2 (3×Ar-*C*), 129.8-127.3 (Ar-*C*H), 124.1 (*C*H-triazole), 61.4 (*C*H_2O), 56.3 (*C*HN), 47.3 (*C*H_2N), 38.2 (*C*H_2NSO_2), 29.7 (*C*H_2), 28.1 (CH_3*C*H_2), 18.4 (*C*H_3CH), 11.1 (*C*$H_3$$CH_2$). Elemental analysis calcd (%) for $C_{29}H_{32}N_4O_4S$: C 65.39, H 6.06, N 10.52; found: C 65.42, H 6.07, N 10.56.

Synthesis of 3-(tosyloxy)propyl benzoate 17

To a solution of 3-hydroxypropyl *p*-toluenesulfonate [4] (800 mg, 3.47 mmol), DMAP (84 mg, 0.69 mmol) and Et_3N (0.72 mL, 5.20 mmol) in 8.7 mL of dry DCM, benzoyl chloride (1.39 mL, 12.05 mmol) was added dropwise at 0°C under inert atmosphere (Ar). The reaction mixture was stirred at RT overnight and then quenched with water and extracted with DCM (3 x 125 mL). The combined organic phases were dried (Na_2SO_4), filtered and concentrated under diminished pressure. The crude was purified by flash chromatography (10:1 n-hexane-EtOAc) using an Isolute Flash Si II cartridge to afford **17** as colourless oil (56% yield). ^{1}H NMR (400 MHz, $CDCl_3$) δ: 7.99-7.88 (m, 2H, Ar-*H*), 7.76-7.71 (m, 2H, Ar-*H*), 7.56-7.52 (m, 1H, Ar-*H*) 7.44-7.38 (m, 2H, Ar-*H*), 7.26-7.23 (m, 2H, Ar-*H*),4.30 (t, 2H, *J*=6.4 Hz, CH_2 OCO), 4.19 (t, 2H, *J*=6.4 Hz,$CH_2$$OSO_2$), 2.15-2.10 (m, 2H, CH_2).

Synthesis of 3-azido propyl benzoate 18

To a solution of **17** (650 mg, 1.94 mmol) in dry DMF (13 mL), NaN_3 (370 mg, 5.7 mmol) was added. The reaction mixture was refluxed at 80 °C for 3h and cooled to RT. Then, 125 mL of $CHCl_3$ were added and the mixture was washed with water (5 x 125 mL). The organic phase was dried (Na_2SO_4), filtered and concentrated under diminished pressure, affording compound **18** without any further purification as a yellow solid (390 mg, quantitative yield).^{1}H NMR (400 MHz, $CDCl_3$) δ: 8.03-8.05 (m, 2H, Ar-*H*), 7.58-7.55 (m, 1H, Ar-*H*), 7.47-7.43 (m, 2H, Ar-*H*),4.43 (t, 2H, *J*=6.4 Hz, CH_2 OCO), 3.49 (t, 2H, *J*=6.4 Hz, $CH_2$$OSO_2$), 2.06 (m, 2H, CH_2).

Supporting material references

1. Nuti E. *et al.* Sugar-based arylsulfonamide carboxylates as selective and water-soluble matrix metalloproteinase-12 inhibitors. *Chem Med Chem.* **11**,1626–1637 (2016).
2. Nuti E. *et al.* Arylsulfonamide inhibitors of aggrecanases as potential therapeutic agents for osteoarthritis: synthesis and biological evaluation. *Eur J Med Chem.* **62**, 379-394 (2013).
3. Wang, J. J., & Yu, W.Anti-Markovnikov Hydroazidation of Alkenes by Visible-Light Photoredox Catalysis. *Chemistry* **25**, 3510–3514 (2019).
4. Moussa, I. A., Banister, S. D., Beinat, C., Giboureau, N., Reynolds, A. J., & Kassiou, M. Design, synthesis, and structure-affinity relationships of regioisomeric N-benzyl alkyl ether piperazine derivatives as sigma-1 receptor ligands. *J Med Chem.* **53**, 6228–6239 (2010).
5. Hay Y., Thon, V., Lau, K., Cai, L., Chen, Y., Mu, S., Li, Y., Peng George Wang, P.G., Chen, X. Highly efficient chemoenzymatic synthesis of β1–3-linked galactosides. *Chem. Commun.* **46**, 7507-7509 (2010).

Figure S1: Inhibition curves for compounds 4c (A, B) and 4d (C). Inhibition of versicanase activity (A, C). ADAMTS-4 (5.5 nM) and -5 (0.4 nM) were incubated either with inhibitor or DMSO for 2 h at 37°C before addition of V1-5GAG (50 nM). At each time point, reactions were stopped by addition of EDTA and ADAMTS-generated versican fragments (versikine) quantified by sandwich ELISA. B) Inhibition of peptidolytic activity. ADAMTS-5 (5 nM) was incubated with compound **4c** for 2 h at 37°C before addition of the QF peptide. The relative proteolytic activity is presented, with 100% activity corresponding to that in the presence of DMSO alone. Data are presented as mean ± SEM (n=3). IC_{50} values are reported in **Table 2**.

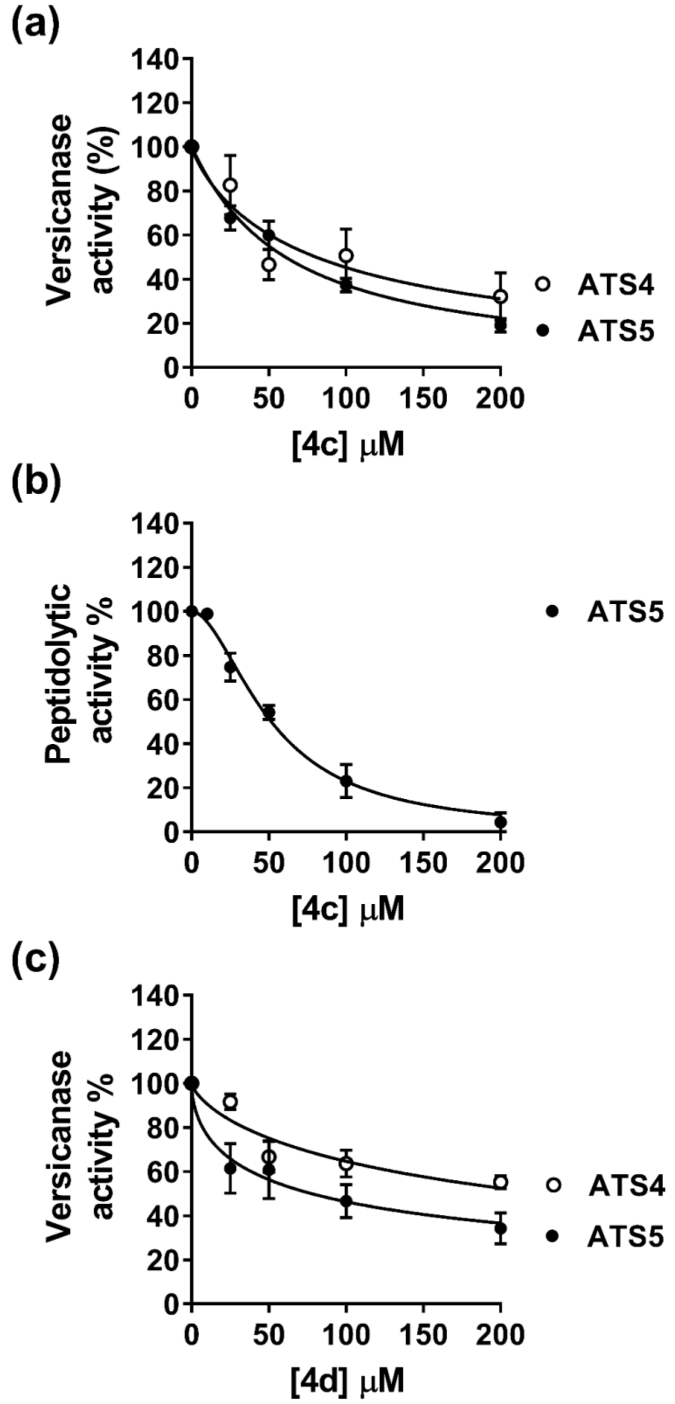

Figure S2: **Representative anti-ARGSV western blot.** Compounds **4b**, **5b** and **6** were incubated with ADAMTS-5 (1 nM) for 2 h at 37°C before addition of aggrecan (20 µg). Following SDS-PAGE and immunoblot, fragments cleaved at the Glu392↓Ala393 bond were detected by a monoclonal neoepitope antibody recognizing the new C-terminal fragment (anti-ARGSV) and analysed by densitometric analysis.

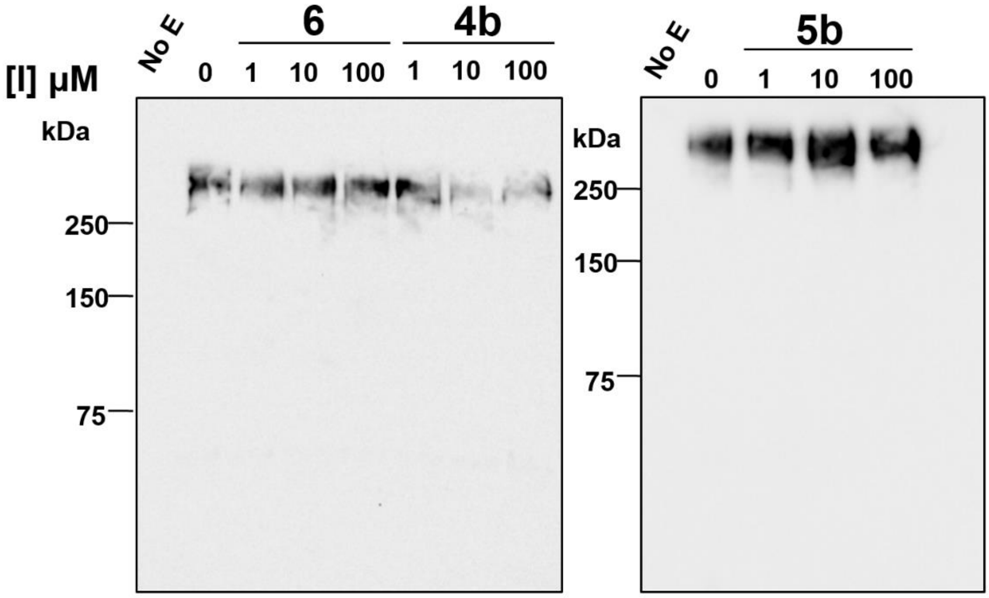

Figure S3: Close-up view of the three binding modes predicted for compound 4b within ADAMTS-5 in the presence of GM6001. The representative binding pose of each cluster (poses C1-C3) is shown. Compound **4b** and GM6001 are shown as green and purple stick models, respectively. The Mp domain is highlighted in gold and the Dis domain in cyan whilst the active site zinc is shown as a grey sphere. Exosite residues K532 and K533 are colored in yellow and purple, respectively. (a) C1; (b) C2; (c) C3.

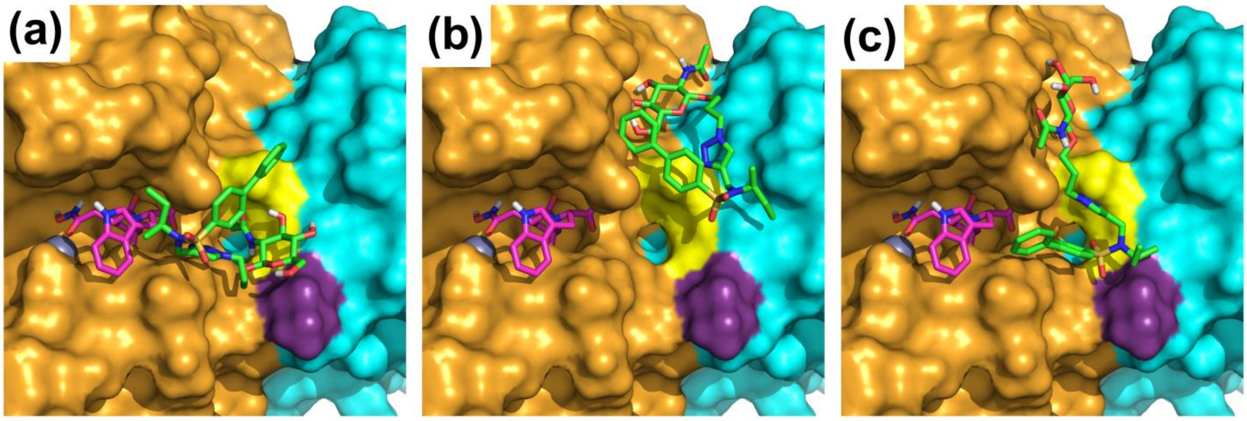

Figure S4. Molecular Dynamics analysis for compound 4b in poses C1-C3. RMSD analysis of the heavy atoms of compound **4b** in poses C1-C3 during the MD simulation.

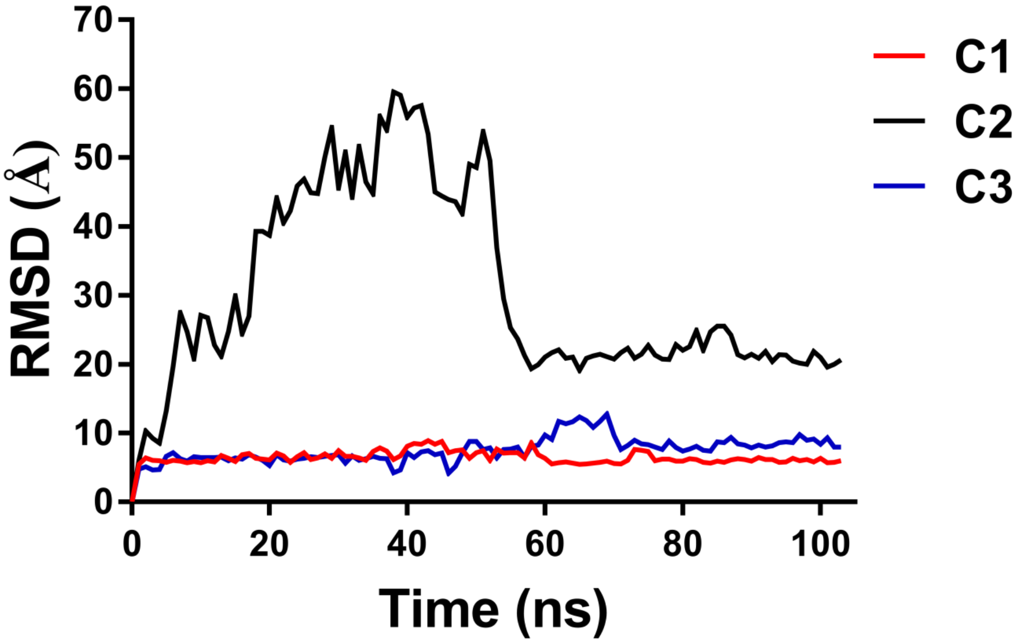

Figure S5: Minimized average structure of the two hypothetical orientations, CL1 (A) and CL2 (B) of compound 4b bound to ADAMTS-5, derived from the last 100 ns of MD simulation. H-bonds are represented as black dashed lines. Compound **4b** is shown as green stick model, whereas the active site zinc is shown as a grey sphere.

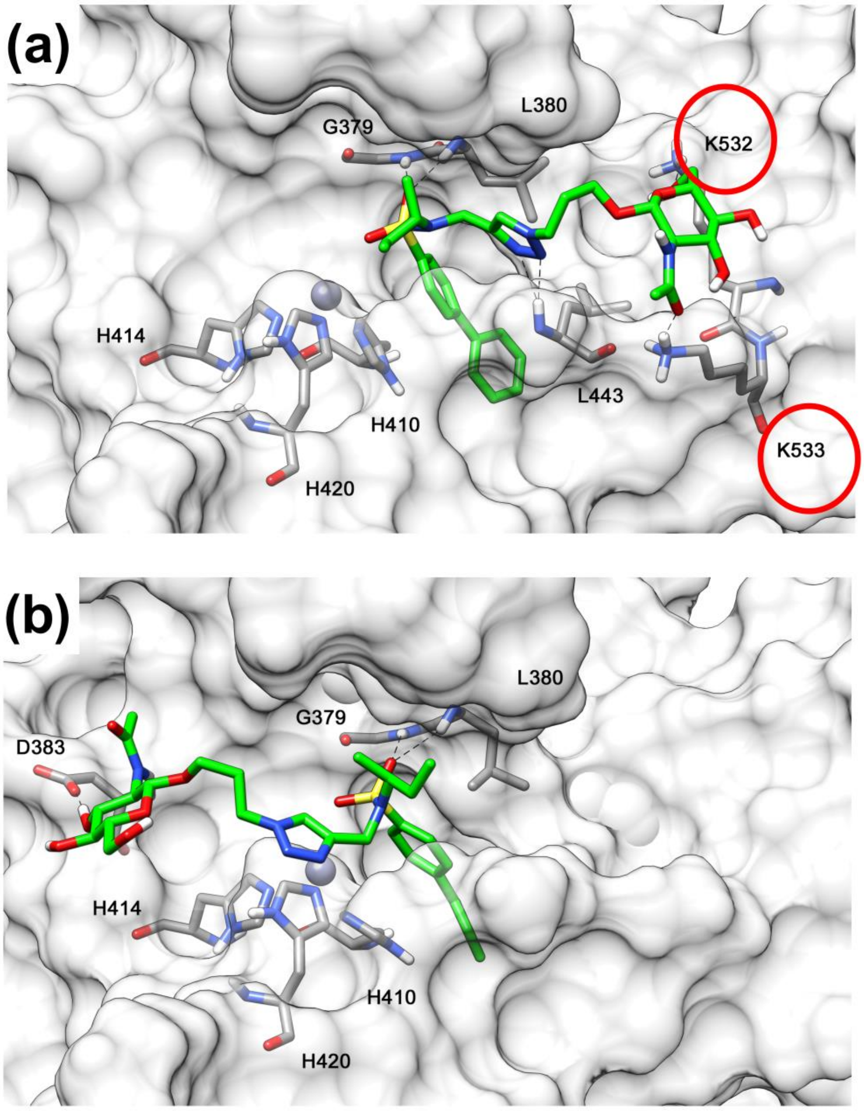

Figure S6: Minimized average structure of compound 4c bound to the ADAMTS-5/GM6001 complex, derived from the last 100 ns of MD simulation. H-bonds are represented as black dashed lines. Compound **4c** and GM6001 are shown as orange and purple stick models, respectively. The active site zinc is shown as a grey sphere.

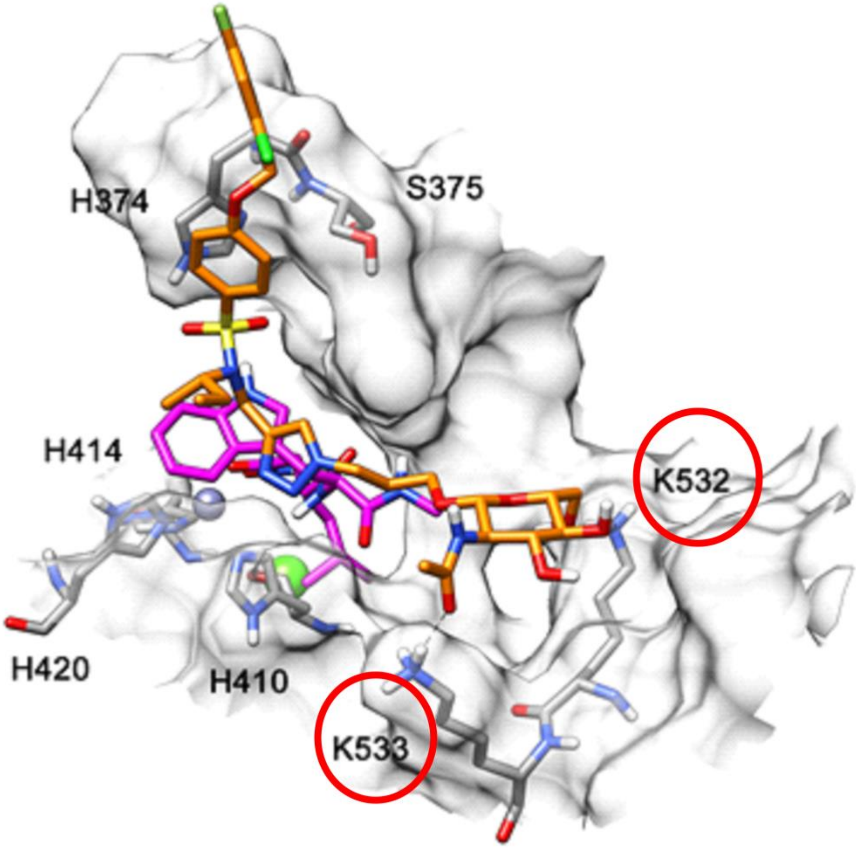

Figure S7: Expression of ADAMTS-5 variants. (A) ADAMTS-5 MDTCS and its Dis domain variants were expressed in HEK293T cells. After 3 days, expression and secretion were analyzed by western blot analysis of conditioned media using an anti-FLAG antibody (Cat. number F1804, Sigma Aldrich). (B) Silver stain of affinity-purified ADAMTS-5 MDTCS and its Dis domain variants. Lanes: 1) wild-type ADAMTS-5 MDTCS; 2) K532A/K533A; 3) K533A; 4) K533H; 5) K532Q/K533Q. Red stars indicate C-terminal processed forms.

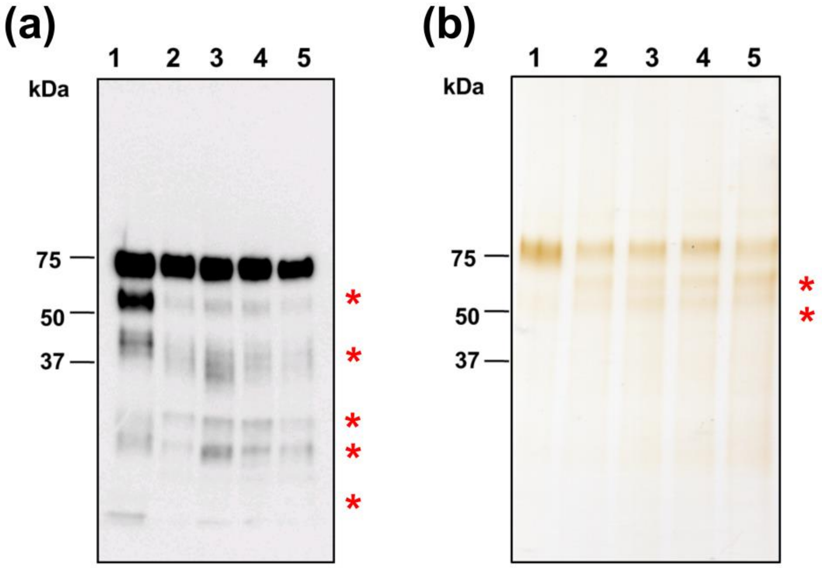

Fig. S8. ^{1}H NMR of compound 3a (400 MHz, CD_3OD).

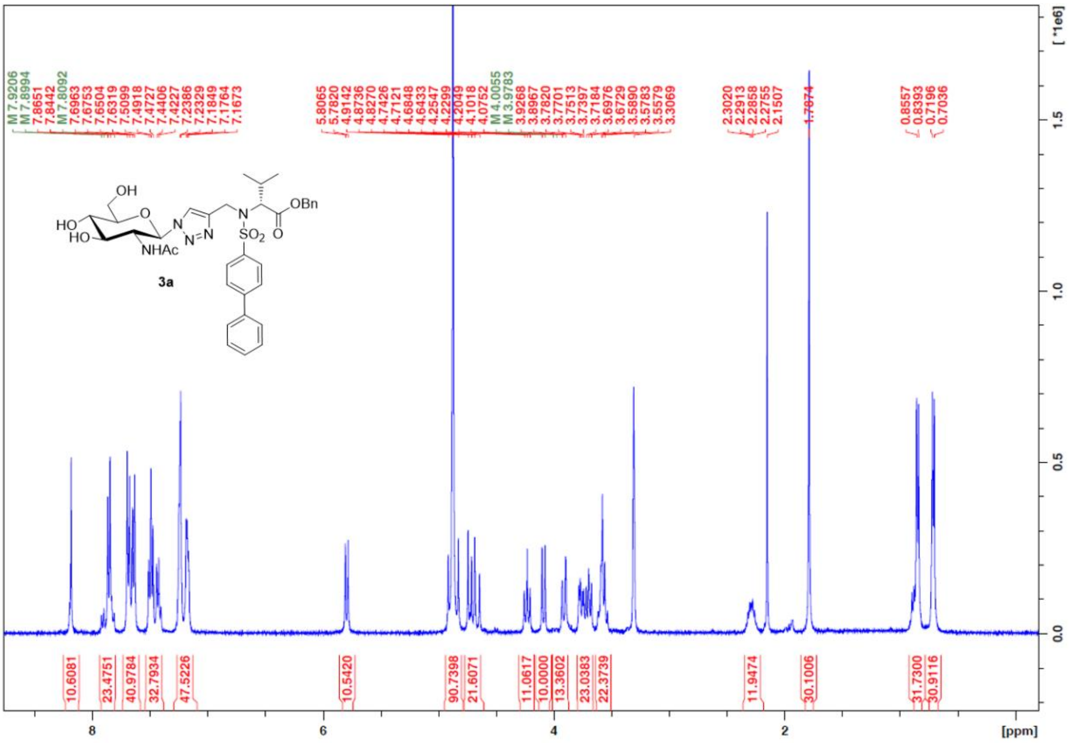

Fig. S9. ^{13}C NMR of compound 3a (100 MHz, CD_3OD).

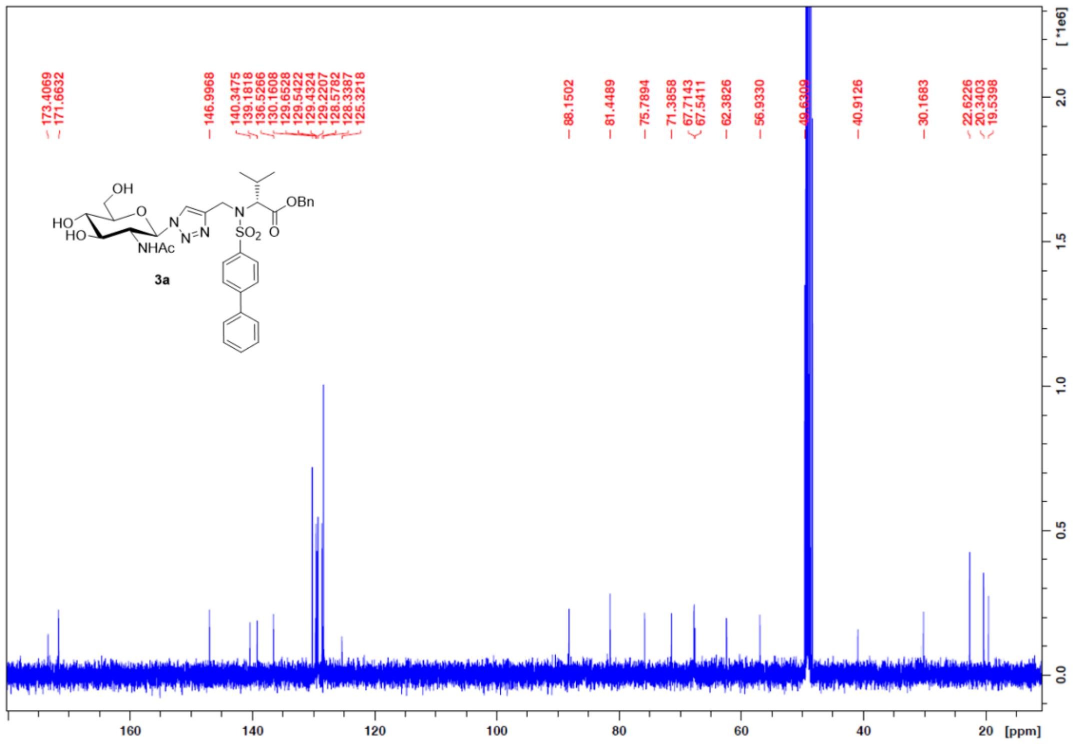

Fig. S10. COSY spectrum of compound 3a (400 MHz, CD_3OD).

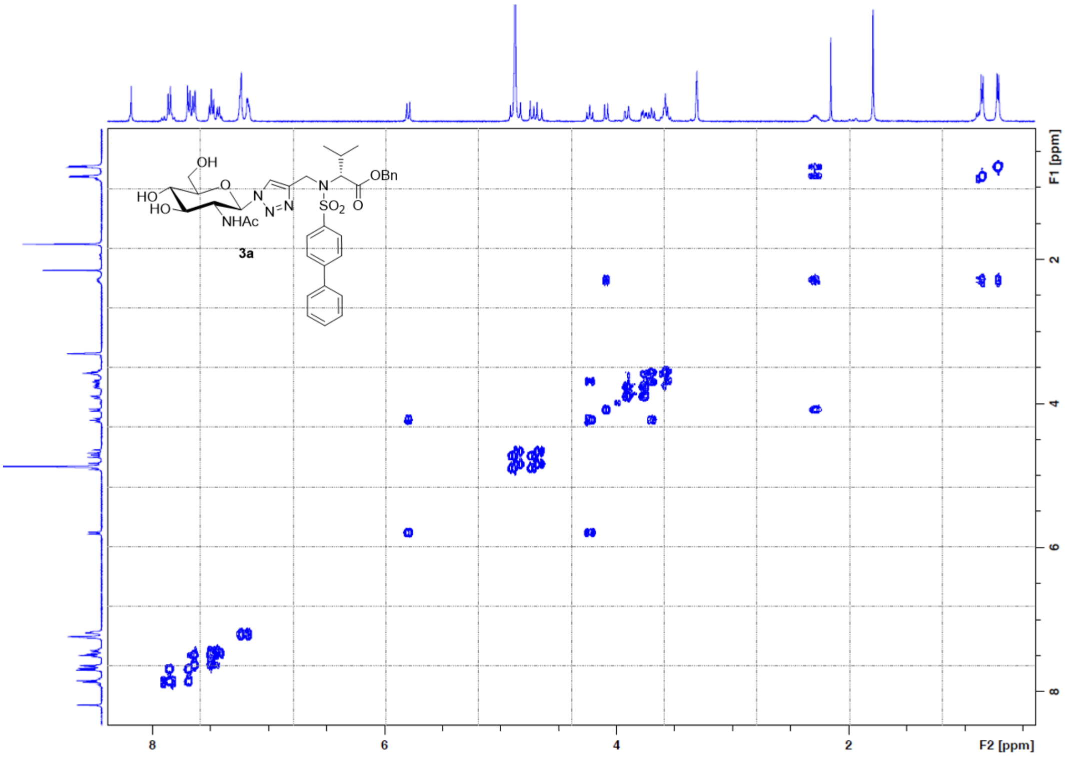

Fig. S11. ^{1}H NMR of compound 3b (400 MHz, CD_3OD).

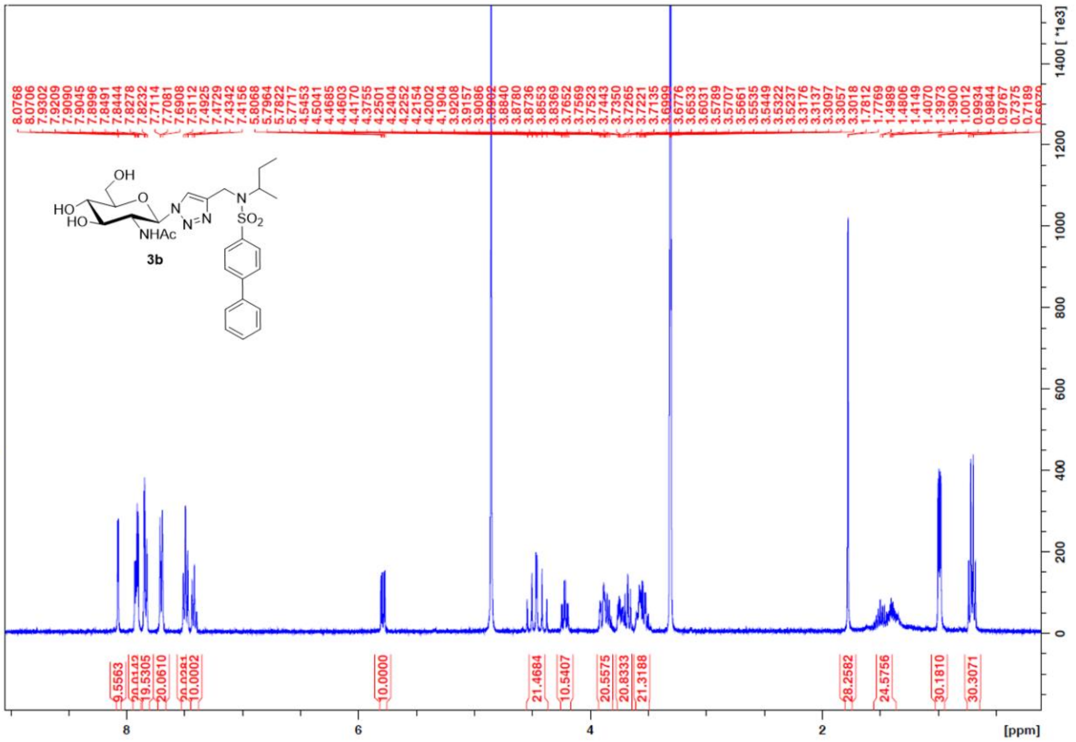

Fig. S12. **^{13}C NMR of compound 3b** (100 MHz, CD_3OD).

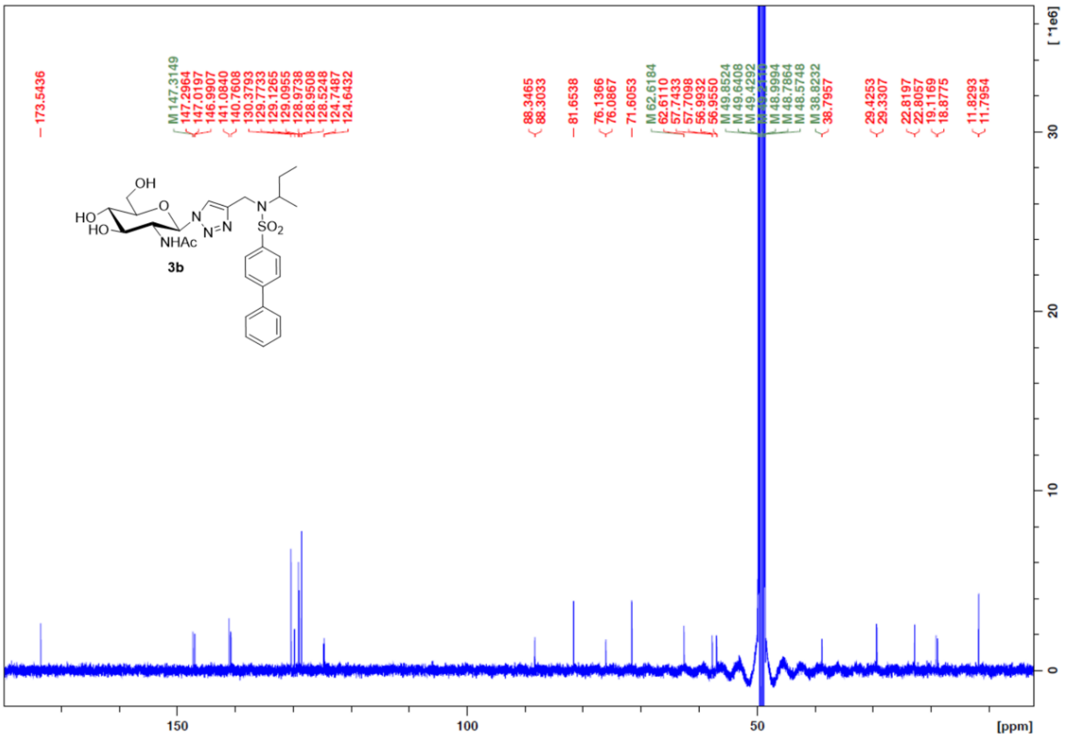

Fig. S13. ^{1}H NMR of compound 4a (400 MHz, CD_3OD).

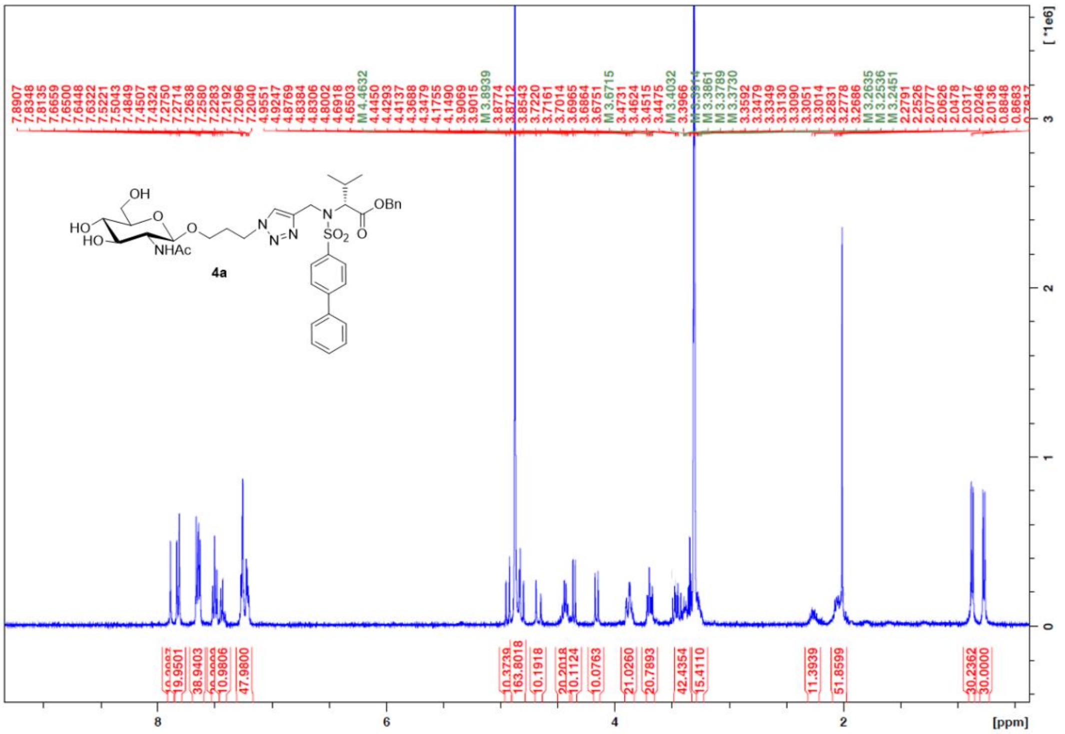

Fig. S14. ^{13}C NMR of compound 4a (100 MHz, CD_3OD).

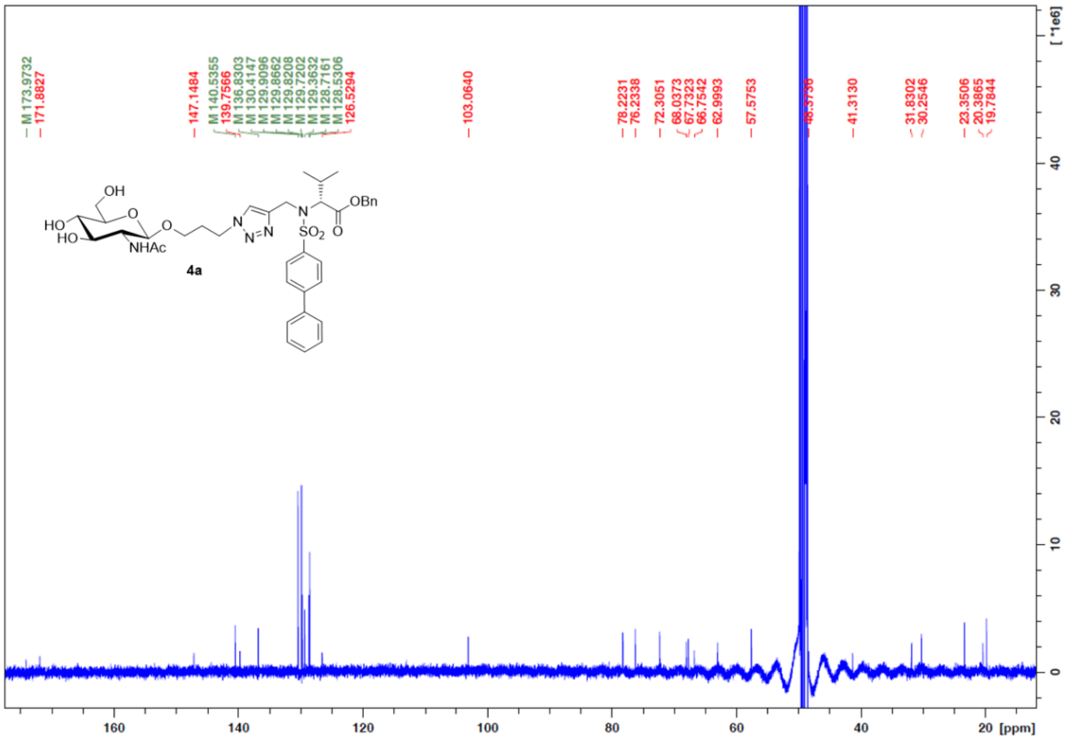

Fig. S15. ^{1}H NMR of compound 4b (400 MHz, CD_3OD).

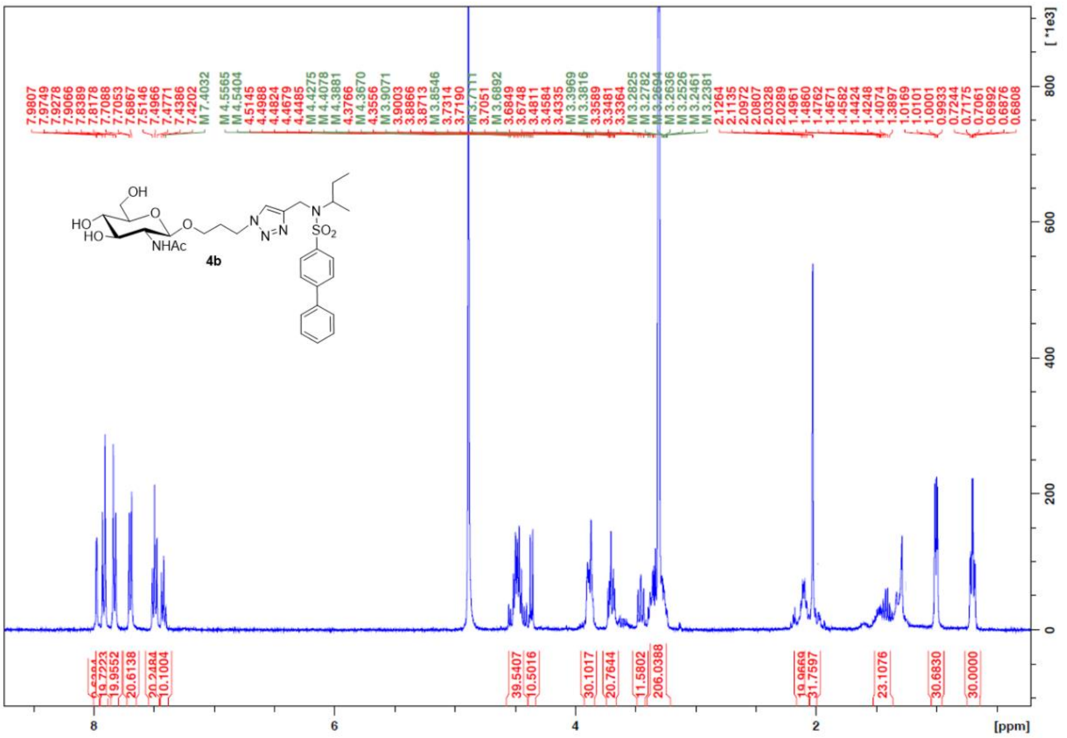

Fig. S16. ^{13}C NMR of compound 4b (100 MHz, CD_3OD).

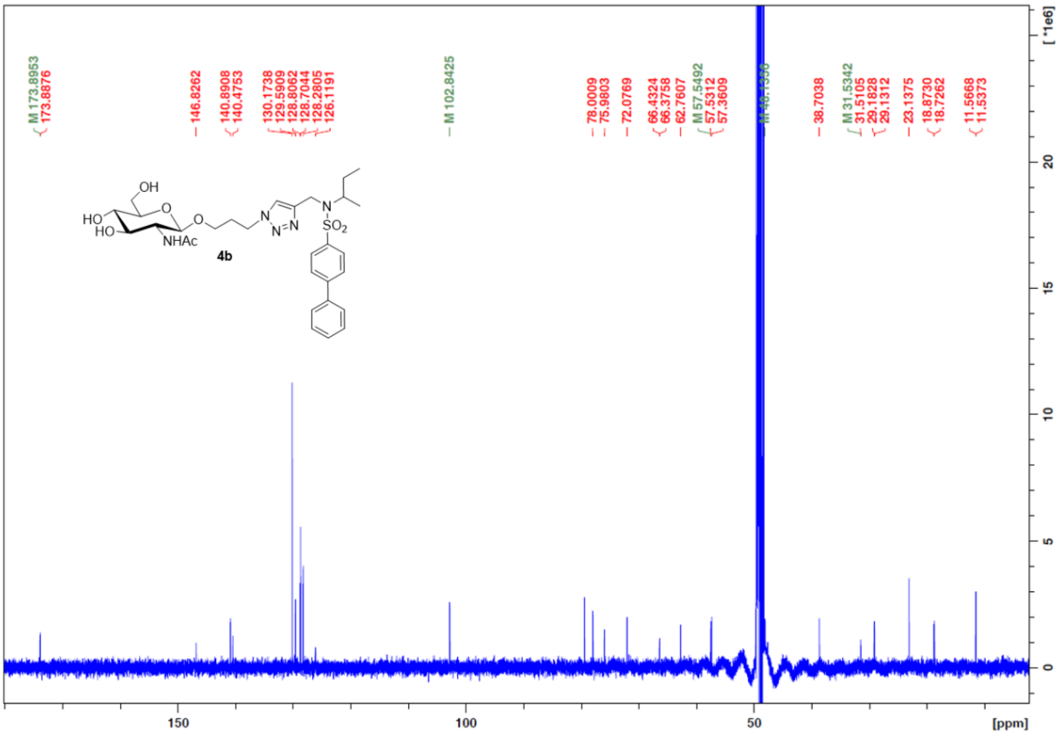

Fig. S17. COSY spectrum of compound 4b (400 MHz, CD_3OD).

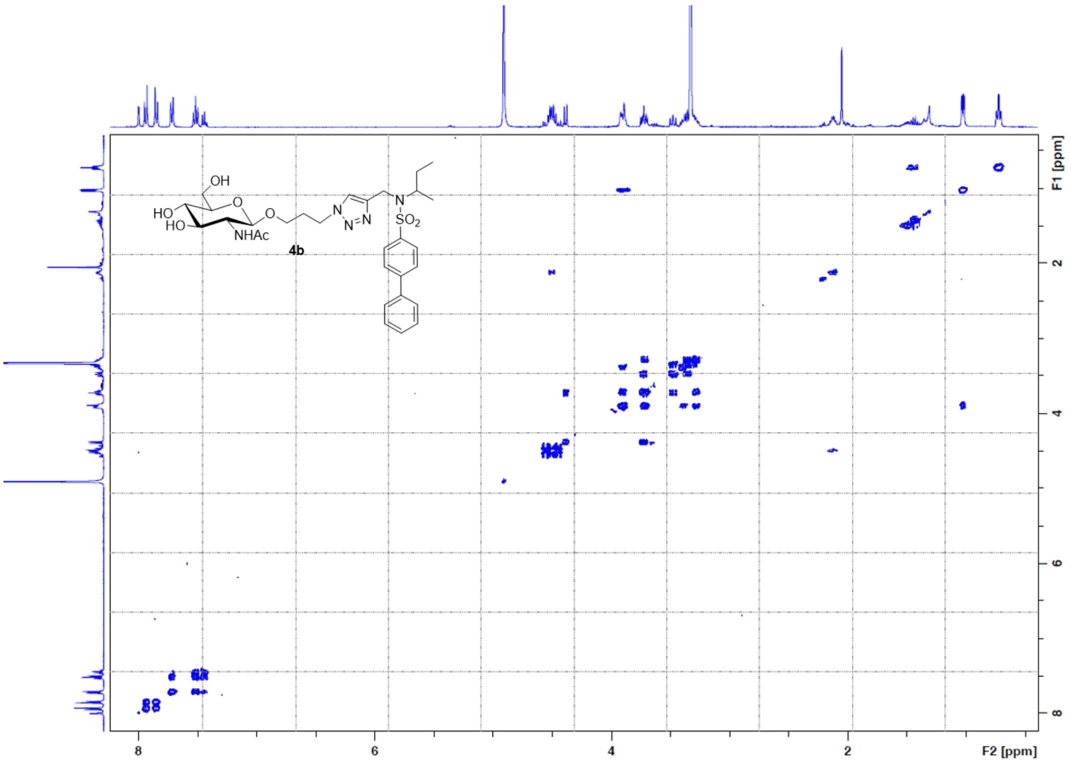

Fig. S18. ^{1}H NMR of compound 4c (400 MHz, CD_3OD).

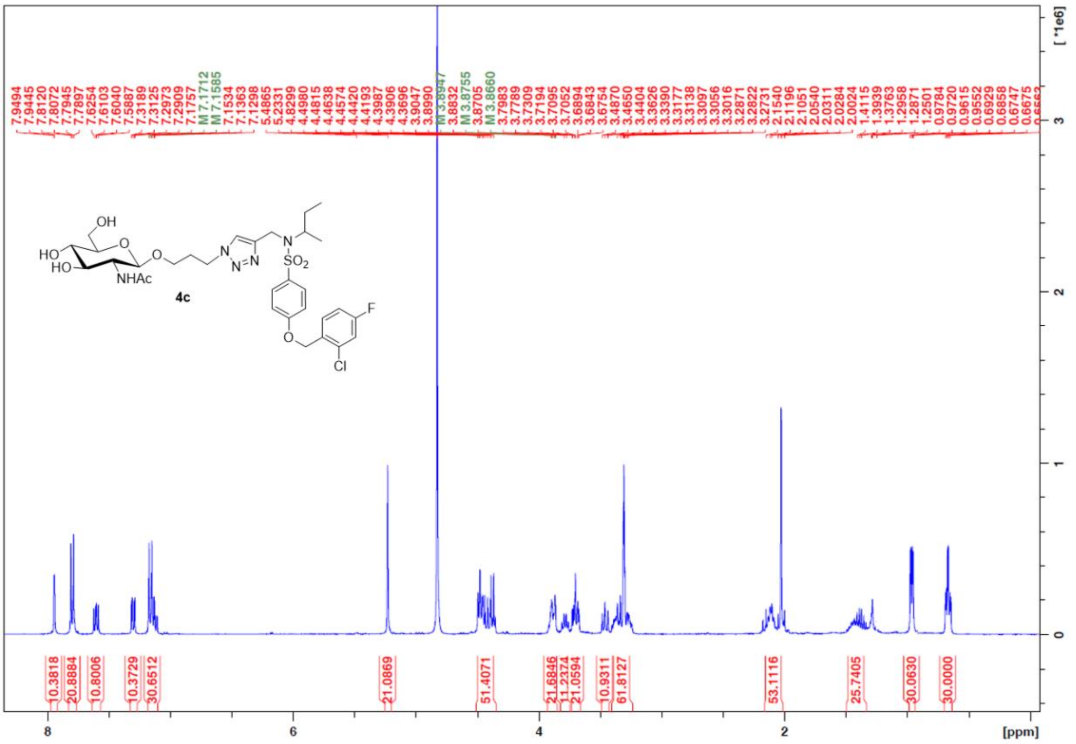

Fig. S19. ^{13}C NMR of compound 4c (100 MHz, CD_3OD).

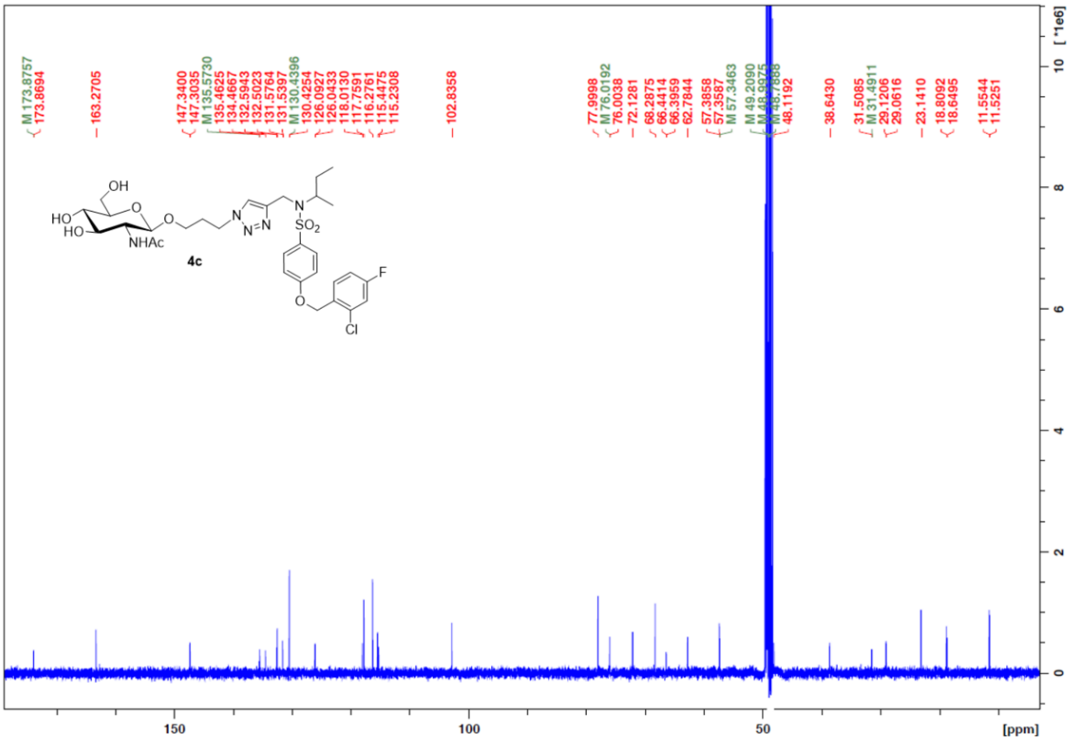

Fig. S20. COSY spectrum of compound 4c (400 MHz, CD_3OD).

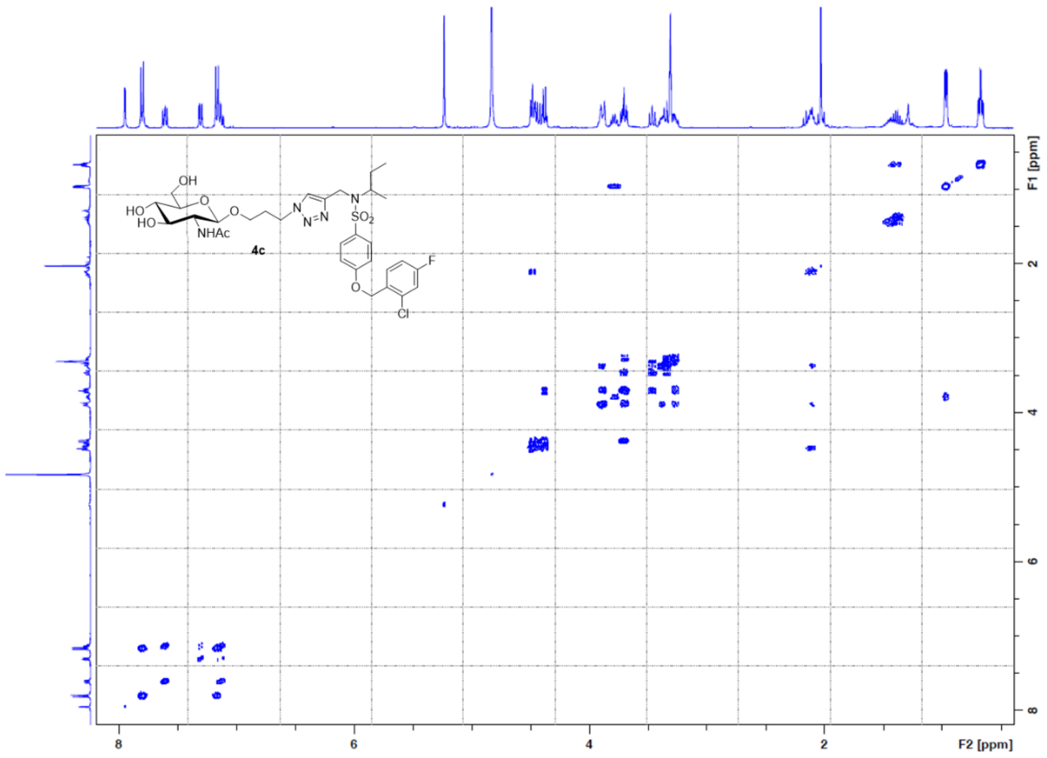

Fig. S21. HSQC spectrum of compound 4c (400 MHz, CD_3OD).

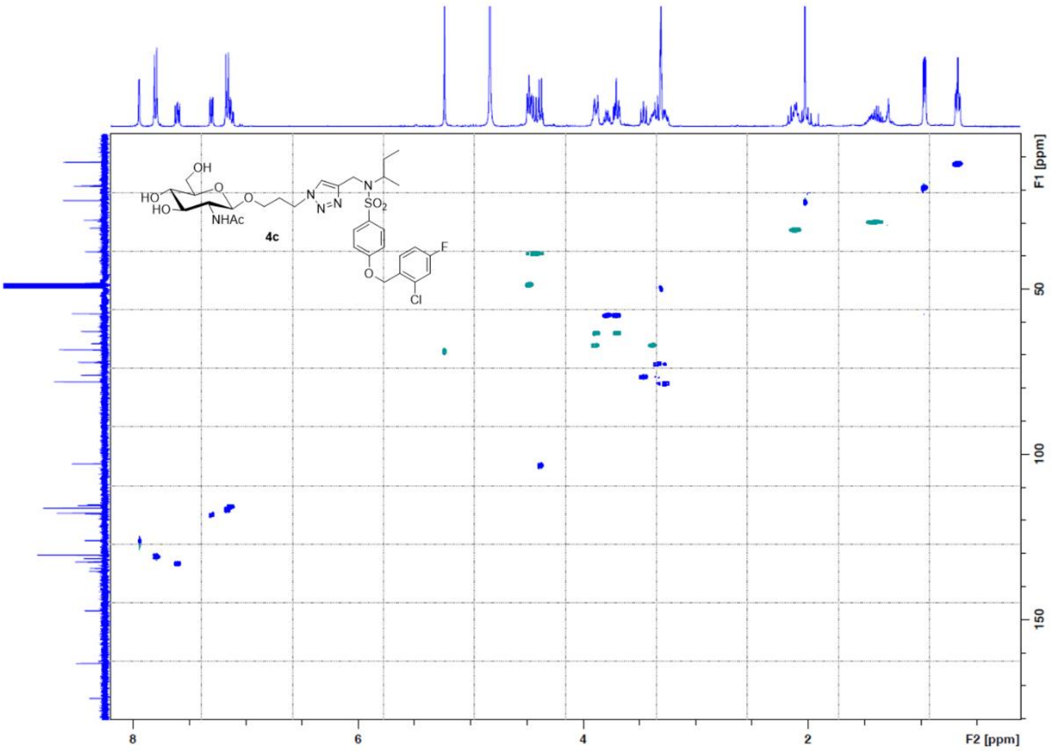

Fig. S22. ^{1}H NMR of compound 4d (400 MHz, CD_3OD).

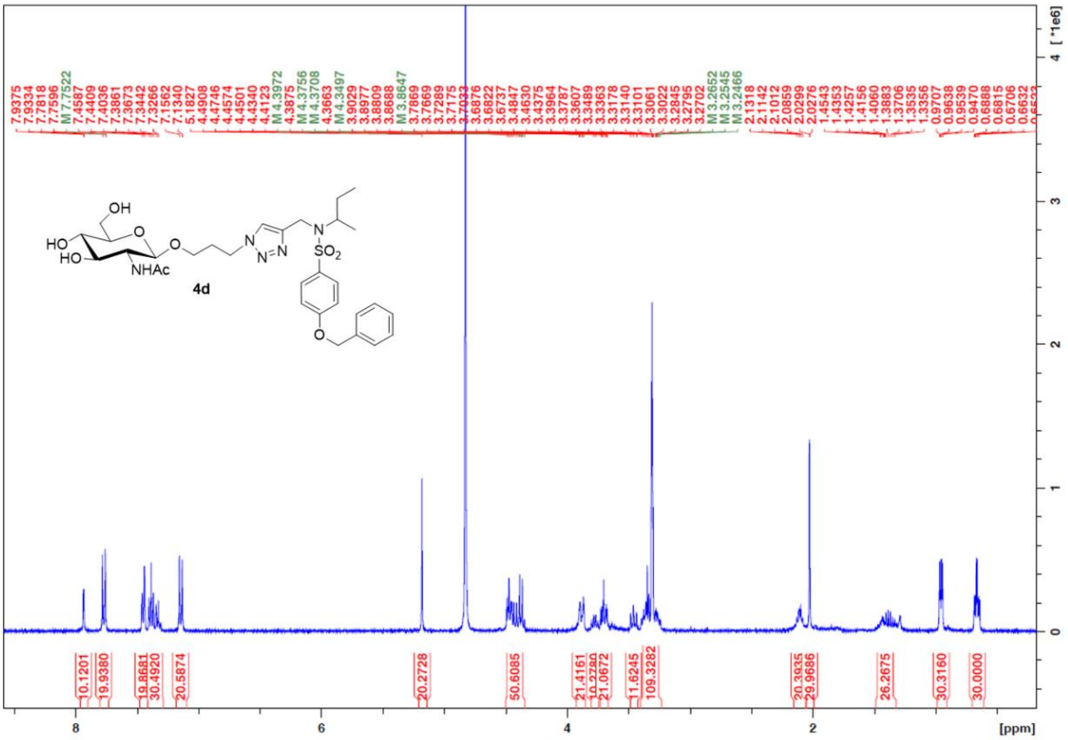

Fig. S23. ^{13}C NMR of compound 4d (100 MHz, CD_3OD).

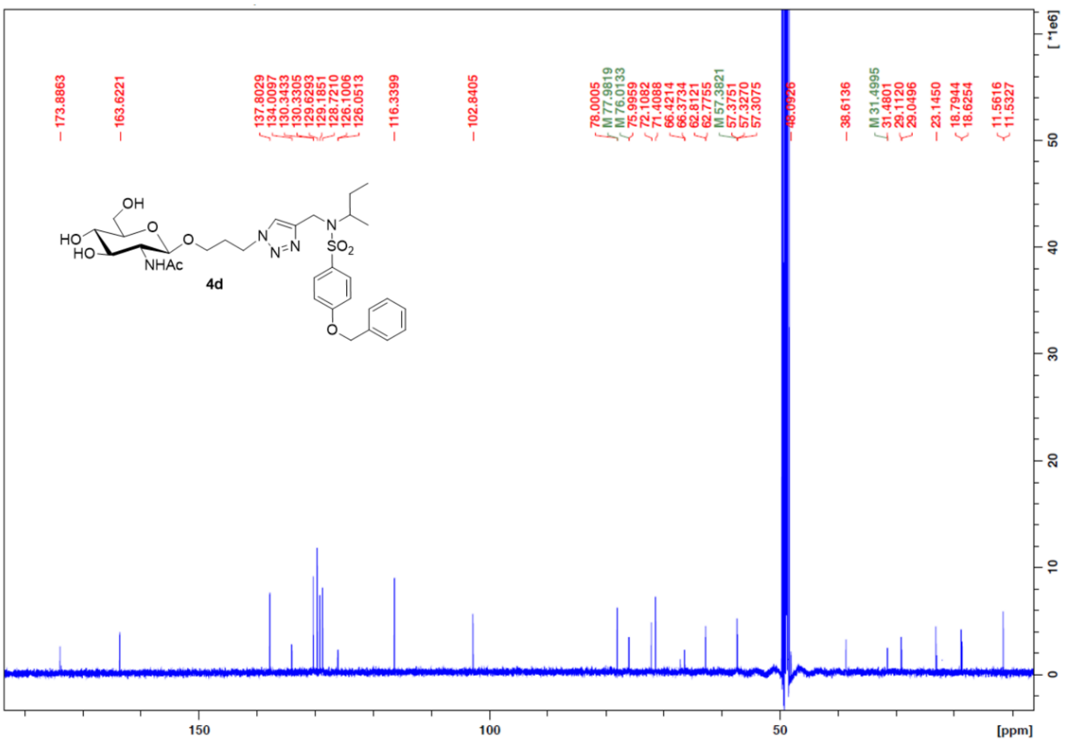

Fig. S24. COSY spectrum of compound 4d (400 MHz, CD_3OD).

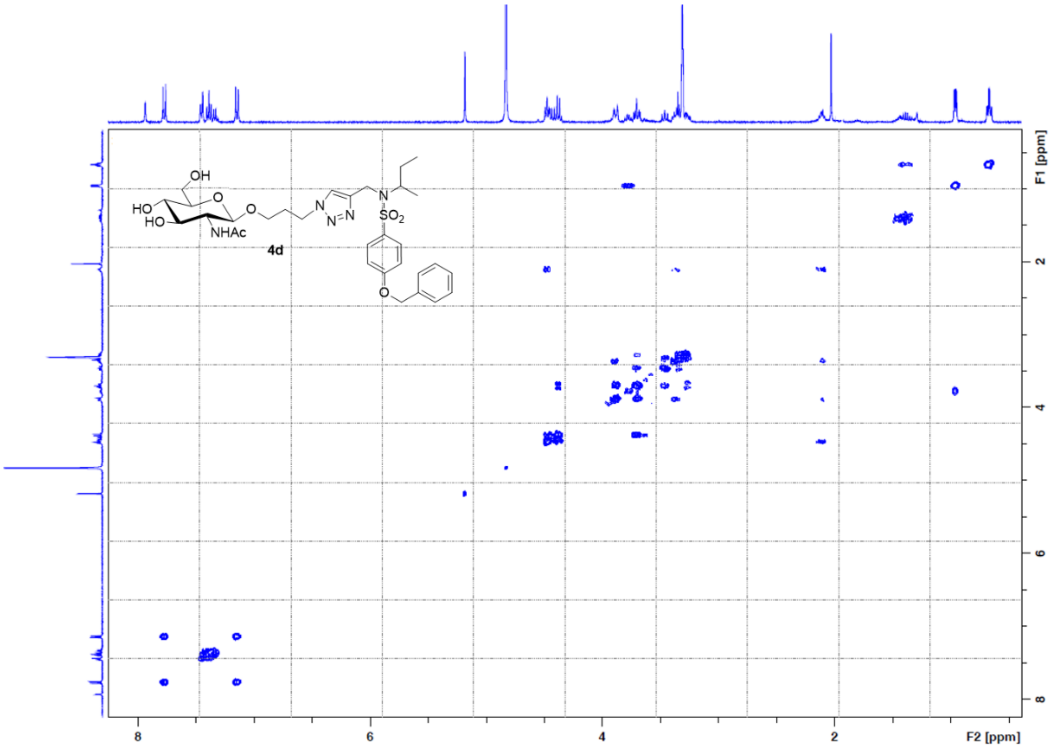

Fig. S25. HSQC spectrum of compound 4d (400 MHz, CD_3OD).

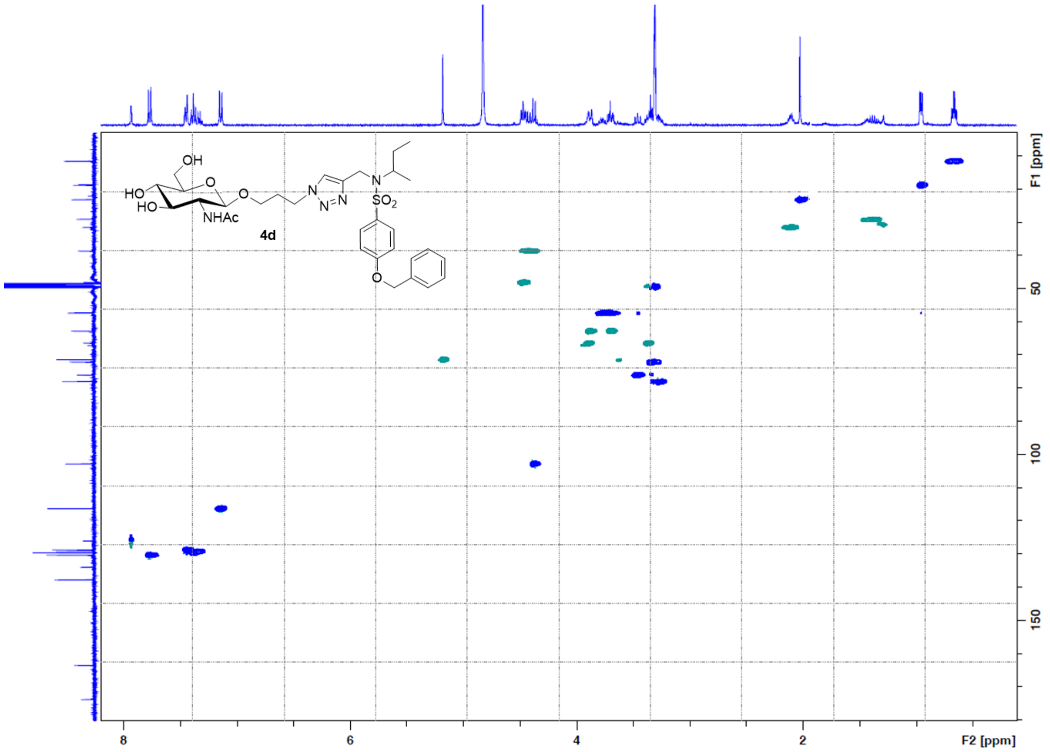

Fig. S26. ^{1}H NMR of compound 5b (400 MHz, CD_3OD).

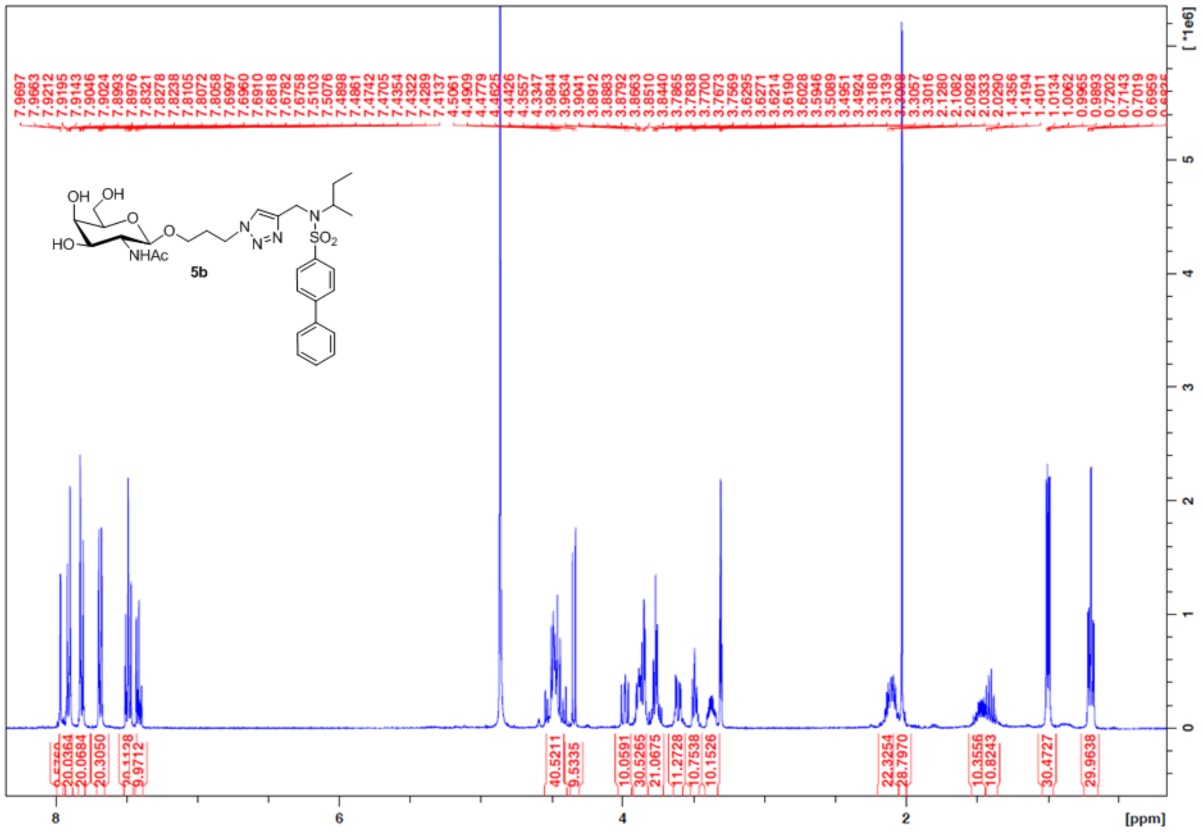

Fig. S27. ^{13}**C NMR of compound 5b** (400 MHz, CD_3OD).

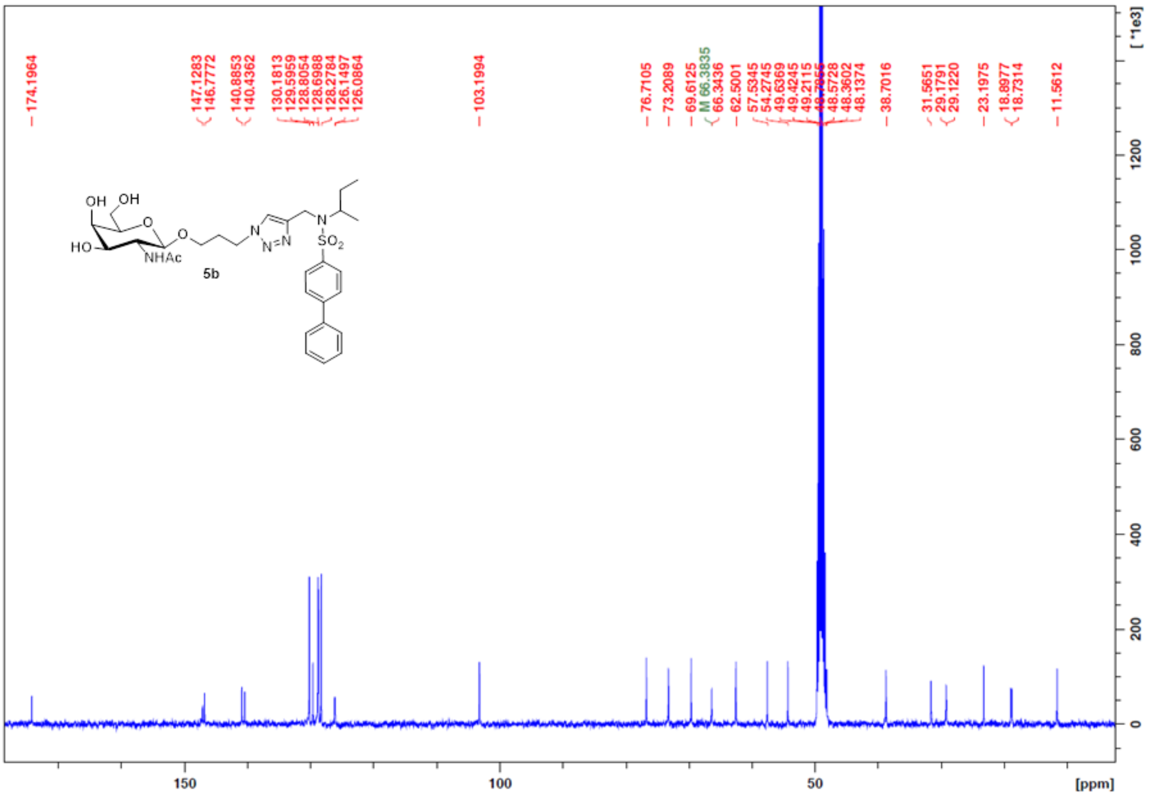

Fig. S28. COSY spectrum of compound 5b (400 MHz, CD_3OD).

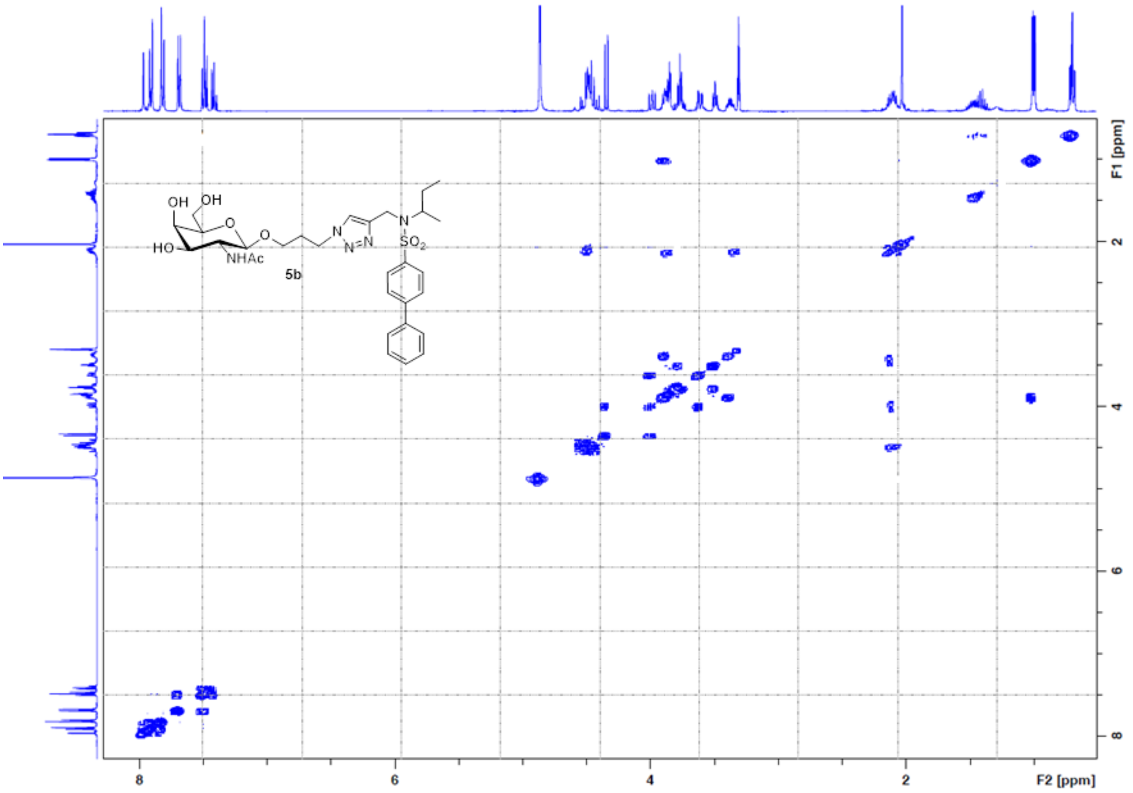

Fig. S29. HSQC spectrum of compound 5b (400 MHz, CD_3OD).

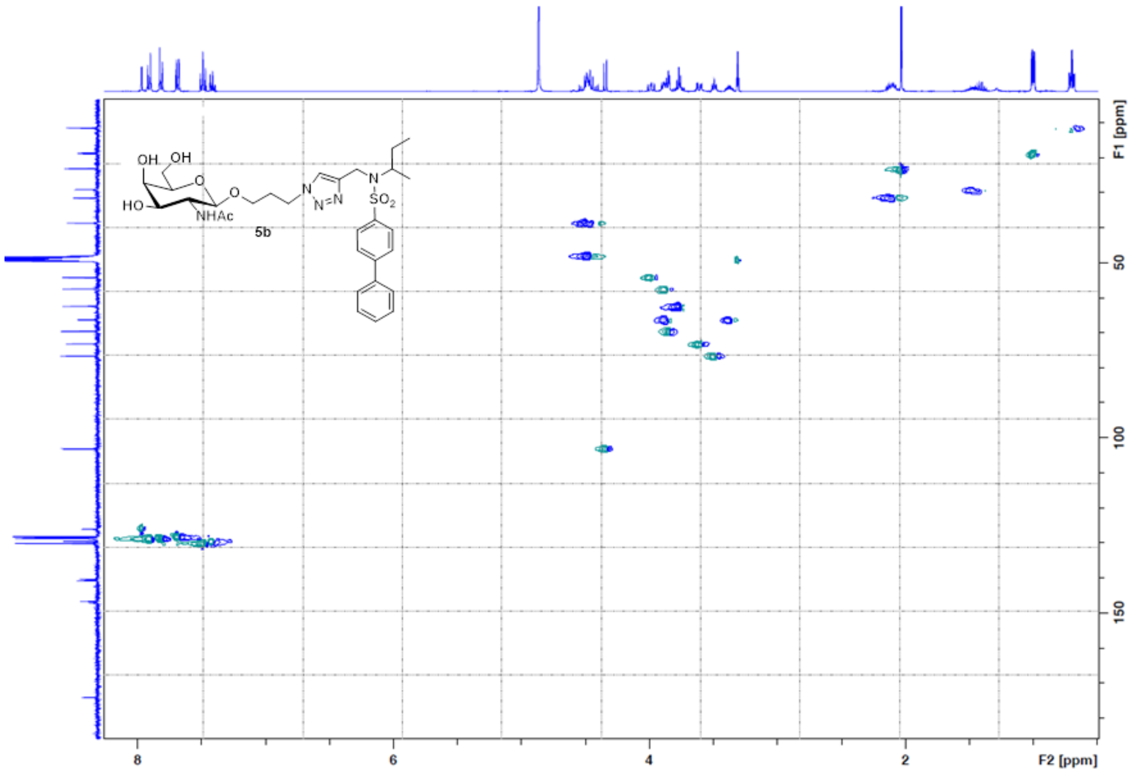

Fig. S30. ^{1}H NMR of compound 6 (400 MHz, $CDCl_3$).

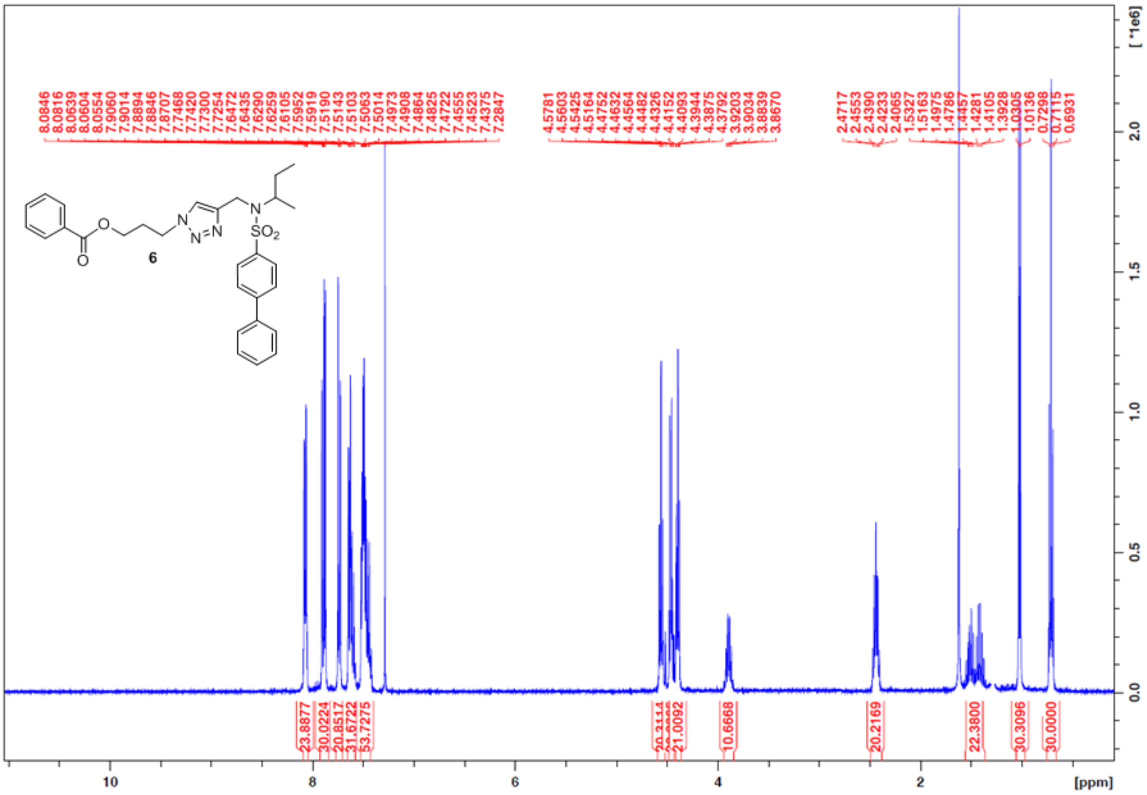

Fig. S31. ^{13}**C NMR of compound 6** (100 MHz, $CDCl_3$).

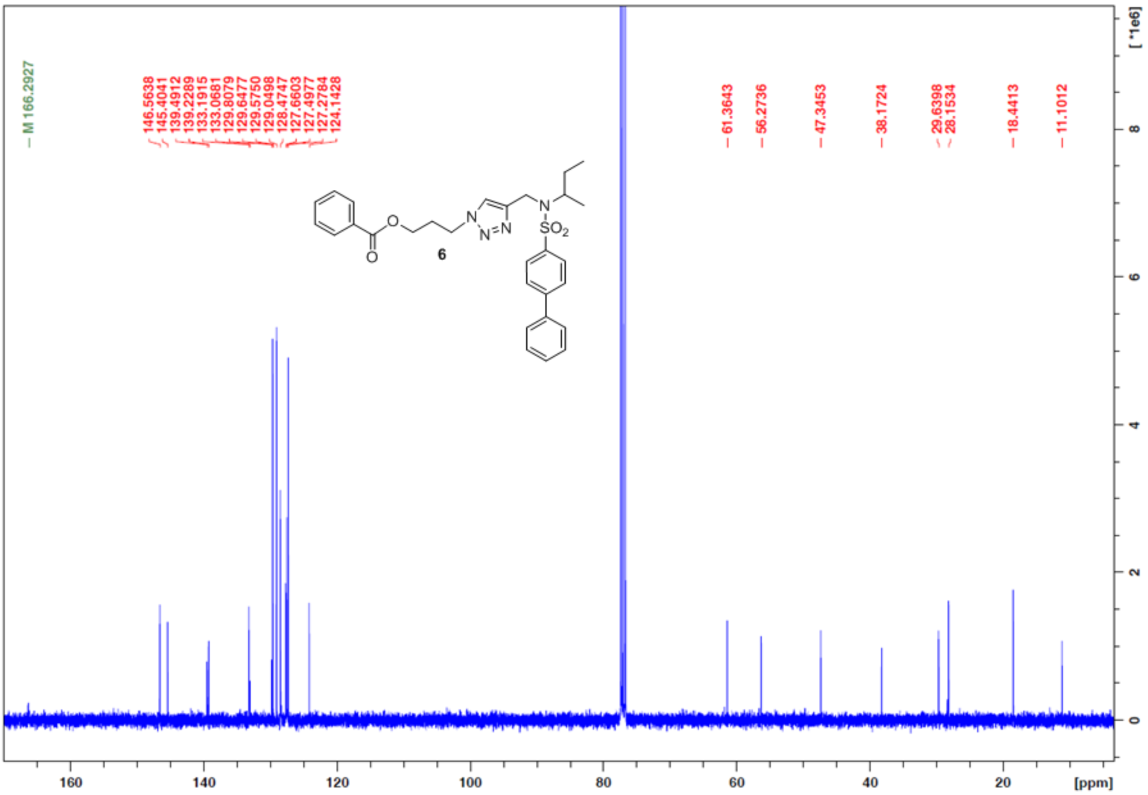

Fig. S32. COSY spectrum of compound 6 (400 MHz, $CDCl_3$).

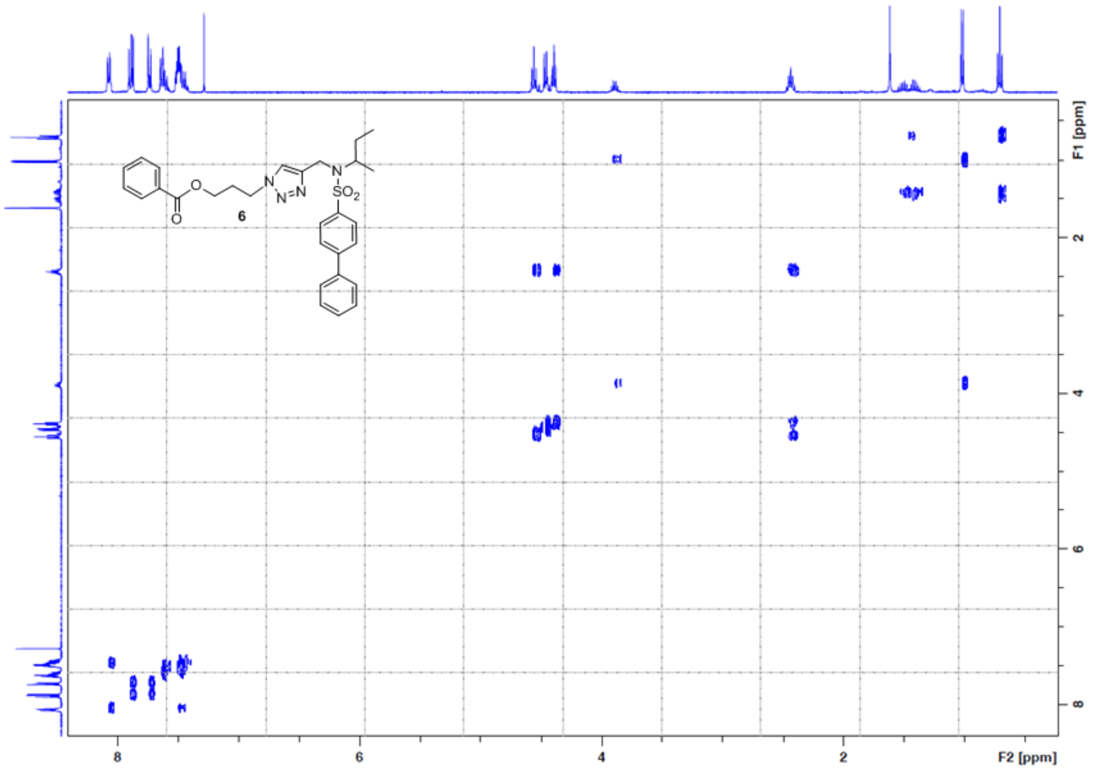

Fig. S33 HSQC spectrum of compound 6 (400 MHz, $CDCl_3$).

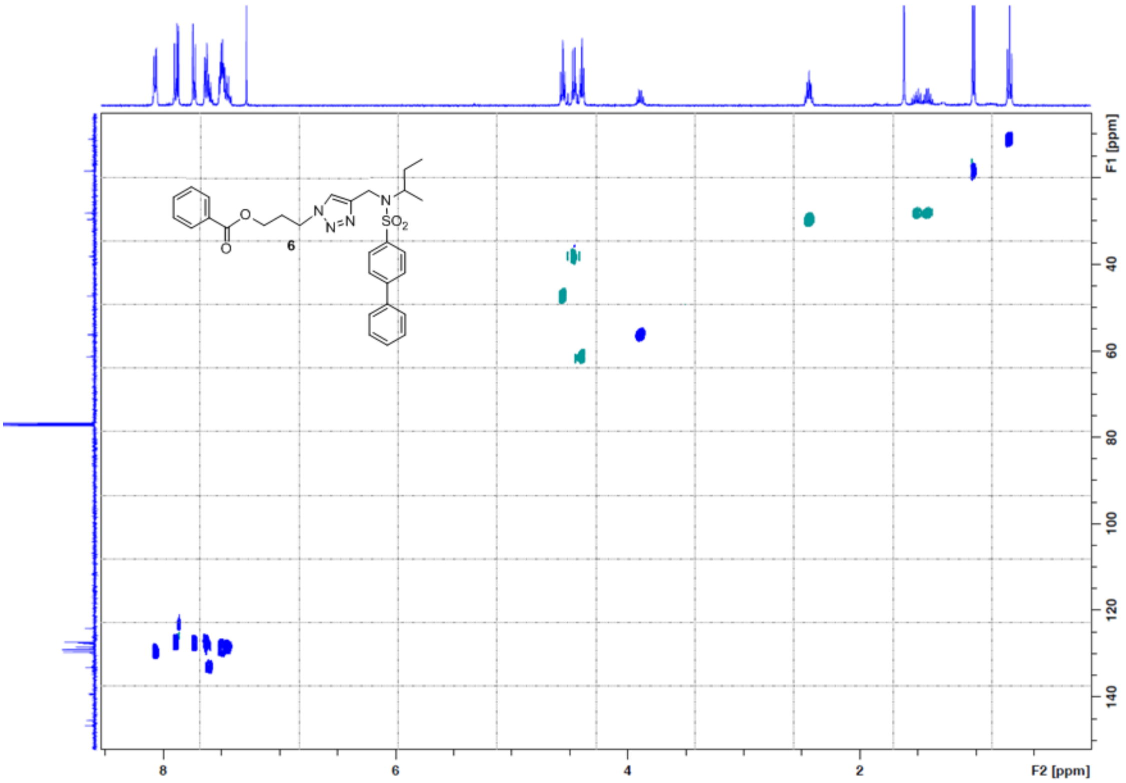

Table S1. MM-PBSA results for the three potential orientations of compound 4b into ADAMTS-5 in the presence of GM6001. ΔPBSA is the total amount of the electrostatic (EEL), van der Waals (VDW), polar (EPB) and non-polar (PBSUR) solvation free energy. Data are expressed in kcal/mol.

MM-PBSA Method					
	VDW	EEL	PBSUR	EPB	ΔPBSA
**C1**	-33.8	-23.8	-3.9	46.7	-14.7
**C2**	-10.8	-5.5	-1.3	12.2	-5.4
**C3**	-30.3	-32.5	-3.8	59.6	-6.9

Table S2. MM-PBSA results for the two potential orientations of compound 4b into ADAMTS-5 in the absence of GM6001. ΔPBSA is the total amount of the electrostatic (EEL), van der Waals (VDW), polar (EPB) and non-polar (PBSUR) solvation free energy. Data are expressed in kcal/mol.

MM-PBSA Method					
	VDW	EEL	PBSUR	EPB	ΔPBSA
**CL1**	-58.0	-43.2	-5.5	80.5	-26.2
**CL2**	-53.2	-32.3	-5.2	62.4	-28.3
